# The Scope of Nurses' Assessment of Deteriorating Patients in Coronary Care Units: A Mixed Methods Study

**DOI:** 10.1111/jocn.17500

**Published:** 2025-01-24

**Authors:** Nicholas Woolfe Loftus, Duncan Smith, Leanne M Aitken

**Affiliations:** ^1^ City St George's, University of London London UK; ^2^ St Bartholomew's Hospital London UK; ^3^ University College London Hospitals London UK

**Keywords:** Coronary Care Unit, Deteriorating Patient, Mixed Methods Research, Patient Assessment

## Abstract

**Background:**

Despite the high acuity of coronary care unit (CCU) patients and their risk of deterioration, little is known about how nurses assess them.

**Aim:**

Increase understanding of the scope of nurses' assessments of deteriorating CCU patients.

**Design:**

Online mixed methods survey.

**Methods:**

The mRAPIDS (modified Rescuing a Patient in Deteriorating Situations) tool was used to measure assessment scope in responses to a patient vignette with a higher mRAPID score signalling broader scope (maximum score 24). Reflections on day‐to‐day practice were collected concurrently and thematically analysed. Themes were integrated with scores using a joint display table and organised into domains. Comparing ‘fit’ between data showed expansion (overlap with broader nonoverlapping findings) and disconcordance (contradictory findings).

**Results:**

Thirty‐four nurses responded, and scope of assessment was found to be narrow (median mRAPIDS 5). Two domains were identified that helped explain this finding ‘the act of assessment’ and ‘education and experience’. Participants emphasised the importance of education and experience, neither increased assessment scope.

**Conclusion:**

This study showed that participant assessments were generally narrower than widely accepted best practice (ABCDE assessment).

**Implications:**

Participant assessments did not reflect gold standard A‐E assessment, which may partly reflect a need for assessment frameworks that are more compatible with real‐world practice. Further research is required to understand the role of healthcare assistants in the care of deteriorating CCU patients. Clinical judgement is important, but not yet well understood in rapid response systems.

**Impact:**

This study offers preliminary understanding of nurses' assessments of deteriorating patients in CCUs.

**Reporting Method:**

American Psychological Association, Mixed Methods Standards.

**Patient or Public Contribution:**

Reviewed protocol, aided result interpretation and shared ideas for future research.

**Summary**

- What does this paper contribute to the wider global clinical community?
  ○ Reports the number and type of clinical cues registered nurses working in coronary care units collect and how these inform their clinical impressions.Identifies the need for further research to deepen understanding of how registered nurses working in higher acuity areas use assessment frameworks and clinical reasoning models in their practice.

## 1 Introduction

Clinical deterioration is the worsening of a patient's physical health resulting in increased risk of morbidity and/or mortality (Jones et al. 2013). Evidence suggests approximately a third of avoidable inpatient deaths arise from suboptimal care of clinically deteriorating patients (Donaldson, Panesar, and Darzi 2014). Suboptimal care encompasses delays in diagnosis, treatment or referral, poor assessment and patient management (Quirke, Coombs, and McEldowney 2011). As well as unplanned ICU admission and cardiac arrest, sub‐optimally managed clinical deterioration is associated with an increased average length of hospital stay (Padilla and Mayo 2019). Consequently, the prevention of identifiable patient clinical deterioration has become a priority area for researchers, clinicians and policymakers around the world.

## 2 Background

Improving care of clinically deteriorating patients requires consideration of acuity which is synonymous with the amount and complexity of care required by a patient. Although a dynamic concept, the categorisation of acuity according to care needs may be clinically useful with Levels 0, 1, 2 and 3 used in the UK context to reflect patient acuity (Department of Health 2000). To bridge the gap between care provided in a ward and a critical care setting, Level 1+ (termed enhanced care) has recently been added to the care continuum (Table 1) (The Faculty of Intensive Care Medicine 2020).

**TABLE 1:** Patient acuity (Department of Health 2000; The Faculty of Intensive Care Medicine 2020).

Level of care (numerical label)	Level of care (description)	Typical location where care provided	Examples of care provided
Level 0	Ward based care	Ward	Vital signs required less frequently than every 4 h Patients requiring intravenous therapy, e.g., antibiotics Often 1 nurse per 6–10 patients
Level 1	Ward based care	Ward	Vital signs required at minimum every 4 h Patients requiring continuous supplemental oxygen therapy
Level 1+	Enhanced care	Ward	Vital signs required every hour or continuous cardiac monitoring Use grew from COVID‐19 response Staffing locally determined
Level 2	High dependency care	Critical care unit	Vital signs required every hour Patients requiring therapies to support **one** organ system, e.g., drugs to support blood pressure Usually 1:2 nurse‐ to‐patient ratio
Level 3	Intensive care	Critical care unit	Patients requiring mechanical ventilation and/or support of **multiple** organ systems, e.g., mechanical ventilation and drugs to support blood pressure Usually 1:1 nurse‐to‐patient ratio

Patients with cardiovascular disease who present to hospital acutely unwell may be cared for in a coronary care unit (CCU). Nursing staff in these specialist areas monitor and provide care to clinically unstable cardiac patients with conditions including (but not limited to) acute myocardial infarction, heart failure and potentially dangerous dysrhythmias (Jones and Johnson 2008). Whilst patient acuity in CCUs can span from level 0 through to level 2 (high dependency) care, these units are often operationally and geographically separate from critical care.

### 2.1 Rapid Response Systems

**FIGURE 1:**
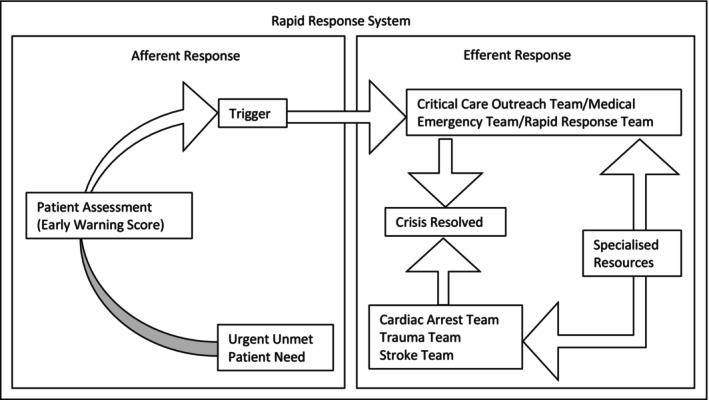
Rapid response system (Adapted from DeVita et al. (2006)).

In the seminal *Critical Care Without Walls* paper, Hillman (2002) argued that critical care should be available throughout hospitals (irrespective of physical location) and proposed a rudimentary rapid response system (RRS) with this aim. RRSs have since been refined and now feature across international guidelines (NICE 2007; American Heart Association 2020; Australian Commission on Safety and Quality in Health Care 2021). These systems are composed of afferent and efferent limbs. The afferent limb involves the identification of clinical deterioration using track‐and‐trigger tools that standardise assessment of patient's vital signs (e.g., heart rate and blood pressure) and prompt clinical staff to call for help (escalate care) when abnormalities in vital signs are detected (Figure 1). The response that follows the escalation of care forms the efferent limb of the RRS (DeVita et al. 2006).

### 2.2 Early Warning Scores

Track‐and‐trigger tools were devised on the premise that a high proportion of patient clinical deterioration (59%) is accompanied by deranged vital signs (Andersen et al. 2016). In the United Kingdom, a specific track‐and‐trigger tool was developed to standardise practice within and between organisations. The National Early Warning Score 2 (NEWS2) is endorsed by NHS England and features in NICE guidance (NICE 2007; Royal College of Physicians 2017). On the NEWS2 chart (now often electronic), vital signs have predefined parameters with higher scores reflecting greater physiological derangement and greater risk the patient will come to harm (Table 2). For specified score thresholds, different protocolised responses are recommended, with lower scores prompting an increased frequency of vital signs monitoring, and higher scores recommending a practitioner from a designated response team with expertise in the care of clinically deteriorating patients (e.g., a Critical Care Outreach Team (CCOT)) be contacted as part of the efferent limb.

**TABLE 2:** NEWS2 scoring parameters (Royal College of Physicians 2017).

Parameter	Score						
	3	2	1	0	1	2	3
Respiratory rate	≤ 8		9–11	12–20		21–24	≥ 25
SpO_2_—scale 1 (%)	≤ 91	92–93	94–95	≥ 96			
SpO_2_—scale 2 (%)	≤ 83	84–85	86–87	88–92 ≥ 93 on room air	93–94 (on oxygen)	95–96 (on oxygen)	≥ 97 (on oxygen)
Air or oxygen		Oxygen		Air			
SBP (mmHg)	≤ 90	91–100	101–110	111–219			≥ 220
Pulse (per minute)	≤ 40		41–50	51–90	91–110	111–130	≥ 131
Consciousness				Alert			CVPU
Temperature (°C)	≤ 35.0		35.1–36.0	36.1–38.0	38.1–39.0	≥ 39.1	

Abbreviations: CVPU, confusion, voice, pain, unresponsive; SBP, systolic blood pressure; SpO_2_, peripheral oxygen saturations.

The predictive performance of NEWS (the precursor to NEWS2 that includes the same vital signs and scoring ranges) has been validated in a range of different level 0–1 (ward) settings (Spångfors et al. 2019) and with prehospital, surgical and septic patient populations (Redfern et al. 2018; Klepstad et al. 2019; Pullyblank et al. 2020). Although NEWS2 outperforms most other track‐and‐trigger systems that are used internationally, false positives and false negatives are reported. Ethnographic research has shown that 42% of nursing staff' afferent limb behaviours did not adhere to NEWS protocols (Smith et al. 2020). Deviant behaviours included inaccurate measurement of respiratory rates, inappropriate delegation of patient assessment to health care assistants, incomplete and delayed documentation, and selective escalation. Furthermore, findings from a review demonstrate non‐compliance with EWS protocols across all key areas including EWS calculation accuracy, monitoring frequency and clinical response (Credland 2018). It appears that despite widespread implementation, nurses' afferent limb behaviours and use of EWSs deviate from protocols. Explanations for these deviations include nursing staff believing that the tool is restrictive and that it does not promote clinical decision‐making (Burke and Conway 2023).

### 2.3 Patient Assessment in Coronary Care Units

As NEWS2 was developed for use in ward settings, there is paucity of research focussing on the validity and usefulness of the tool to nurses working in level 1+ and 2 areas that are independent of critical care (including CCUs). One of the challenges of using tools like NEWS2 in these areas is the potential for false positive alerts. From an observational study conducted in a higher care area in Holland, 19 false positive alerts were reported for every true deterioration event (Plate et al. 2018). These findings highlight the potential limitations of using these tools in higher acuity areas and underscore the potential importance of broader clinical assessments.

Although tools like NEWS2 were designed to standardise the assessment of clinically deteriorating patients, sensitivity may be improved if more information is included (Smith et al. 2020). Whilst the use of these tools is not meant to preclude the incorporation of information beyond vital signs (Royal College of Physicians 2017), their use may encourage nurses to focus solely on vital signs rather than interpreting them alongside other clinical information (e.g., urine output and peripheral skin temperature) (Jensen, Skår, and Tveit 2018; Chua et al. 2019). Recognition that NEWS2 can unintentionally diminish clinical assessment led to a multicentre, cross‐speciality testing of a clinical assessment add‐on to NEWS: Individual EWS (I‐EWS) (Nielsen et al. 2022). In the intervention group, I‐EWS allowed nurses to adjust the score up or down (or not) based on their clinical assessment. For all‐cause mortality at 30 days, I‐EWS was found to be non‐inferior to NEWS. These findings suggest that the integration of clinical assessment findings alongside an objective score preserves patient safety whilst potentially reducing false positive alerts.

### 2.4 Rationale

It is well established that patient assessment is an important part of CCU nurses' professional role given the acuity and clinical instability of the cardiac patients they care for (Jones and Johnson 2008), and the potential for more abrupt deterioration without the usual antecedent derangement in vital signs detectable using track‐and‐trigger tools (Alhmoud et al. 2023). Despite the importance, there is a lack of research focusing on how CCU nurses assess deteriorating patients and how NEWS2 is integrated into their assessment and subsequent decision‐making. More broadly, the role and suitability of the RRS in areas that are not classified as wards but fall outside the usual structures of critical care (including CCUs) has not been established. CCU nurses' assessments of clinically deteriorating patients, and our understanding of it, are limited, and further research is needed to improve care and inform policy. The research question driving this research was What is the scope of registered nurses' clinical assessment of deteriorating patients in CCUs?

## 3 The Study

### 3.1 Aim

To increase understanding of the scope of nurses' assessment of deteriorating CCU patients.

### 3.2 Objectives

1. Measure the scope of nurses' assessment of a deteriorating CCU patient.
2. Explore nurses' perceptions of their assessment scope using a clinical vignette.
3. Through integration of findings, deepen understanding of nurses' scope of assessment.

## 4 Methods

### 4.1 Design

**FIGURE 2:**
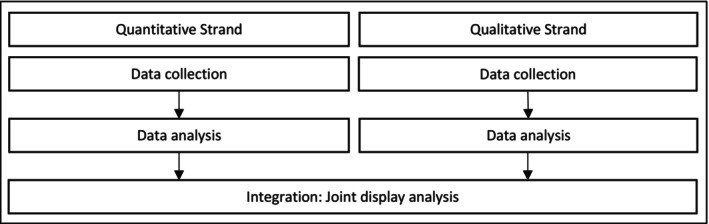
Design.

A convergent parallel mixed methods design was used in which quantitative and qualitative data were collected concurrently from each participant (allowing direct comparison), analysed separately and then integrated using a joint display approach (Figure 2). The quantitative methodology was explorative and qualitative descriptive. The use of a mixed methods approach enabled quantitatively measured scope to be better understood through participants qualitative perceptions (Polit and Beck 2020). As this study sought to generate new knowledge, an inductive approach was used in which hypotheses were generated as opposed to being tested (Polit and Beck 2020). American Psychological Association (2020) Mixed Methods Standards guided detail and transparency of reported content.

### 4.2 Methodology

From a mixed methods perspective, the study used a pragmatic approach where the aims of the study determined the qualitative and quantitative methods. Pragmatism rejects traditional modes of enquiry favouring approaches that are best suited to answering the research question (Polit and Beck 2020). It is commonly used within mixed methods research because it enables simultaneous use of qualitative and quantitative research methods and is orientated towards real‐world practice (Creswell and Plano Clark 2018).

Qualitative data analysis was inductive, in which coding and theme development was driven by the data. The use of an experiential approach ensured that analysis was grounded within the data, rather than more interpretative. Ontologically, analysis was conducted from a simple realism position in which existence of an objective reality was assumed. With this perspective, qualitative analysis predominantly focussed on explicit surface level meaning of the data, which ensured analysis aligned with the aim to understand nurses' disclosed perceptions.

### 4.3 Recruitment

Recruitment was through professional, academic and social channels. Three recruitment strategies were used: The British Association for Nursing Cardiovascular Care (BANCC) (the only UK association for cardiac nurses) distributed recruitment emails to its members (*n* = 211) (File S1). The course lead at City St George's, University of London also distributed emails to postgraduate cardiac nursing course students (*n* = 20). Recruitment advertisements were placed on social media (Twitter) with a quick response code so interested nurses could access the study (File S2).

A sample size of 30 participants is recommended for this type of mixed‐method research (Creswell and Plano Clark 2018). Samples of this size are a balance between manageable amounts of qualitative data and quantitative power and generalisability. Voluntary response sampling was used as recruitment was online through email and social media and because it is cost effective. The inclusion and exclusion criteria for the study were presented at the beginning of the survey for potential participants to self‐determine if they were eligible to proceed.

#### 4.3.1 Participants

To maximise reach whilst balancing time constraints, the recruitment phase was 2 months (December 2022 to February 2023). Three reminder emails were sent, and social media posts were reposted.

The inclusion criteria were as follows:

- Registered nurse;
- Working in England;
- Employed on a CCU either now or within the last 6 months. The 6‐month window was a pragmatic decision to avoid excluding nurses from participating who may have relatively recent relevant experience;
- Agenda for change NHS bands 5–8a; and
- Substantive, agency or bank.

The exclusion criteria were as follows:

- Unregistered nursing staff (e.g., student nurses, health care assistants and nursing associates).

A study‐specific stakeholder group was also assembled: a cardiology registrar and a CCOT physiotherapist, and a group of three patient advisors with relevant lived experience (recruited via an engagement team). The group's contributions were broadly:

1. Reviewing a summary of the research protocol;
2. Participating in a discussion meeting to aid in the interpretation of the results and reviewing the finished study; and
3. Discussing future research.

### 4.4 Data Collection

All data were collected using a Qualtrics online survey, with open‐ended qualitative and closed quantitative questions interwoven on one page (see File S3). Avoiding separate pages reduced the risk of survey fatigue. For the qualitative strand, participants used free text to respond to open‐ended questions asking them to reflect on aspects of their day‐to‐day practice, including their approach, confidence, improvements and feelings (File S3). These questions were developed using published literature (Smith et al. 2021) and through discussion and debate with the entire research team and clinical members of the stakeholder group.

In the quantitative strand, participants were shown a brief vignette of a deteriorating CCU patient (File S3). Vignettes are useful in exploring sensitive and challenging situations in healthcare (Tremblay et al. 2022). To avoid pre‐empting participants' answers, an open‐ended question was used asking how they would assess the patient and if they required any further information. Intentionally, the vital signs provided were only those necessary to calculate NEWS2. The patient in the vignette had early signs of deterioration (e.g., they were tachypnoeic), but only had a low NEWS2 (an aggregate score of 3 where the maximum is 20) to encourage participants to consider the broader clinical information required.

Qualtrics display logic enabled the provision of further information to participants depending on the answers they typed. For example, if they had typed any common variant of ‘electrocardiogram’ or ‘auscultation’, they were then provided with an image of an electrocardiogram or an audio clip of breath sounds. The use of images and audio also helped enhance the vignette's fidelity (i.e., the level of realism). Participants were asked for their interpretation of any further information provided.

Participants were then shown basic information about calculating and responding to a NEWS2 that is usually readily accessible to nurses in practice and asked for their impression of the patient and how they would respond. Demographic questions were asked last. Quantitative data were exported from Qualtrics into SPSS Version 28 for analysis.

The survey was piloted with three members of the Acute and Critical Care Research group at City St George's, University of London (a multi‐disciplinary group of clinicians and academics with backgrounds in acute, cardiac and critical care settings). Piloting confirmed the useability of the survey on different devices and informed improvement of survey content and layout (see File S4 for specific examples).

### 4.5 Research Materials

With Liaw et al.'s (2011) permission, the RAPIDS (Rescuing a Patient in Deteriorating Situations) tool was modified (mRAPIDS) to quantitatively measure the scope of participants' assessments of CCU patients (File S5). Modifications were informed by national guidelines for clinical assessment of deteriorating patients (Resuscitation Council UK 2021). mRAPID items were evaluated by members of the research team (NWL, DS, LMA) and stakeholder group using content validation index (CVI) testing. The number of mRAPIDS items was reduced to 24 by removing:

- Arterial blood gas interpretation (CVI 0.6)
- Palpate chest wall (CVI 0.6)
- Tracheal deviation (CVI 0.4)
- Signs of haemorrhage (CVI 0.2)
- Pupil size and reactivity (CVI 0)
- Temperature (CVI 0.67)

With the mRAPIDS tool, a score of 1 was assigned to items of assessment mentioned in participants' answers and 0 to those not. Participants' interpretations of further information were dichotomised into correct (scored 1) and incorrect (scored 0) using criteria developed with the research team.

Answers to the impression and response questions were initially dichotomised with criteria into correct/incorrect. When developing the criteria, impressions from two randomly selected cases were independently checked by members of the research team (NWL and DS) for agreement (level of agreement was initially 50%). Discordant views about what constituted a correct impression were reconciled through discussion and debate. After initial analysis, it became clear that dichotomising impression answers into just ‘correct/incorrect’ did not accurately represent the data. A third ‘partially correct’ category was therefore added before a further two cases were randomly selected for independent review. Subsequently, researchers had complete agreement in whether the participant's impression of the vignette was correct, partially correct or incorrect.

Researchers (NWL and DS) independently applied the mRAPIDS checklist to two questionnaires where participants had given relatively expansive answers. A comparison of their answers produced a Kappa of 0.738 for participant 5 and 0.865 for participant 17. Differences were discussed and wording of the checklist amended accordingly. Subsequent comparisons of participants 2 and 12 produced Kappa's of 1.

### 4.6 Data Analysis

After familiarising himself with the qualitative data, NWL coded the data independently. Codes were developed by identifying connections and congruence within participants' answers. Coded data were scrutinised and then organised accordingly into clusters bound by shared meaning. Next, theme names were carefully developed to represent the shared meaning within each cluster.

Themes were reviewed against the raw data and relationships between themes considered. Final refinement was a lengthy iterative process, in which other researchers (DS and LMA) checked themes, codes and exemplar verbatim quotes. Feedback from this process informed the adjustment of domain and theme labels to represent the subordinate data more accurately. These data were then reported with verbatim quotes followed by a unique participant identifying code (P1‐34) and then ‘B’ followed by a number denoting their band.

Quantitative data were reported descriptively using medians (with interquartile ranges) and analysed using appropriate non‐parametric statistical tests. Mann–Whitney *U* tests were used to examine differences between two groups, and Kruskal–Wallis tests were used for three or more groups. When other tests have been used, they are reported beside the relevant result. Using joint display analysis, the domains were constructed from common qualitative themes. Quantitative findings of relevance were incorporated into each domain and the ‘fit’ between qualitative and quantitative data in each domain was compared, and meta‐inferences extrapolated.

### 4.7 Ethical Considerations

Ethical approval was obtained through the City St George's, University of London Nursing Proportionate Review Committee (reference: ETH2021‐0423). Partway through recruitment, minor ethics amendments were obtained to expand recruitment to other social media platforms (e.g., Facebook and LinkedIn) and to increase the sample size to mitigate slow recruitment and the brevity of responses from some participants. Participants accessed the study through a weblink or quick response code in recruitment emails and social media posts. Before being presented with the vignette, participants needed to confirm they had read the embedded participant information and given consent to proceed.

Steps were taken to ensure participant anonymity. BANCC and City St George's, University of London distributed recruitment emails on behalf of the research team, which avoided the team having potential participants' email addresses. After consenting, participants were asked if at the end of the study they wanted a results summary and only those who did were asked to provide their email. Participants were reassured that if they provided their email, they would be taken to the study through an anonymous link, so it would be impossible to link their email with their answers. It was explained that email addresses would be securely stored on the GDPR (General Data Protection Regulation) compliant platform Qualtrics. City St George's, University of London also has an agreement in place with Qualtrics to ensure requisite information governance protection.

## 5 Results

This mixed methods study was designed to understand nurses' assessment of deteriorating CCU patients through an online survey. Thirty‐six CCU nurses participated in the study and provided responses, although two were < 50% complete and therefore discarded, leaving a final sample of 34. Most respondents were recruited from social media (Twitter = 20, Facebook = 4, LinkedIn = 3), for whom it was not possible to calculate a response rate due to the lack of a denominator. Of all BANCC members 1% responded (4 from 211), and 10% of City St George's, University of London students (2 from 20). One participant did not specify how they were recruited. The survey had a 36% completion rate.

### 5.1 Sample Characteristics

Most participants were staff nurses or junior sisters/charge nurses and were substantively employed (Table 3). The majority had more than 5 years of experience and worked in district general and tertiary hospitals (see Table 3 for further details about all characteristics).

**TABLE 3:** Joint display analysis table.

Domains	Qualitative themes	Quantitative results	Meta‐inferences
The act of assessment	Nurses use and perceived limitations of NEWS2 Nurses deliberately, safely, and empathetically tailoring their actions The stress nurses feel and its perceived impact Temporal dimensions of nurses' assessments	mRAPIDS item frequency: 1–28 (see Figure 3) mRAPIDS median score 5 (IQR 4–8) Moderate signals that higher mRAPIDS score associated with greater likelihood of forming a correct patient impression Median mRAPIDS scores were significantly higher for those who would review documentation	Expansion^a^
Education and experience	Nurses' belief in the importance of education and experience	Median mRAPIDS scores were not significantly different across employment or education	Disconcordance^b^

^a^ Qualitative and quantitative data overlap centrally as well as a broader nonoverlapping interpretations (Fetters 2020).

^b^ Qualitative and quantitative data lead to conflicting interpretations (Fetters 2020).

### 5.2 Qualitative Themes

Qualitative themes were grouped into two common domains: *the act of assessment* and *education and experience* (Table 4). Themes in the act of assessment domain were *nurses' use and perceived limitations of NEWS2*, *nurses deliberately, safely and empathetically tailoring their actions*, *the stress nurses feel and its' perceived impac*t, and the *temporal dimensions of nurses' assessments*. The education and experience domain had a single theme, *nurses' belief in the importance of education and experience* (themes are reported with subordinate codes and exemplar verbatim quotes in Files S6 and S7).

**TABLE 4:** Patient impression statistical analysis.

	Correct impression (*n* = 2)	Partially correct impression (*n* = 11)	Incorrect impression (*n* = 21)	Analysis
mRAPIDS	Md 13.5	Md 7	Md 5	Only correct & incorrect sig. diff. (*p* = 0.015)
mRAPIDS	Combined correct groups (*n* = 13) Md = 7		Md 5	Combined & incorrect groups sig. diff. (*p* = 0.035)

Abbreviations: Md., median; Sig. diff., significant difference.

#### 5.2.1 Themes Within the Act of Assessment Domain

##### 5.2.1.1 Nurses' Use and Perceived Limitations of NEWS2

Junior nurses' descriptions of assessment often conveyed an over reliance on NEWS2 despite the low score in the vignette, whereas senior nurses' responses strongly questioned the suitability of NEWS2 in the CCU context:

Perform observations and frequency based on NEWS. (P6, B5)

… NEWS (is) wholly unsuitable for patients in a CCU environment… (P33, B7)

##### 5.2.1.2 Nurses Deliberately, Safely and Empathetically Tailoring Their Actions

Some participants described a range of actions that they would integrate into their assessment when appropriate, which demonstrated deliberate tailoring of the assessment for an individual patient.

Depending on clinical scenario‐oxygen/NIV/I can perform ABG (extended role), patient positioning, IV access/bloods. Cardiac monitoring/ECG. Ensure patient is in a monitored bed space so patient is visible. (P27, B6)

For some, patient safety was ensured through the tailoring of practice to their own level of competence.

I'm also not afraid to ask questions, to understand rationale for decisions, to aid learning from experiences. (P2, B5)

For other participants, consideration of the patients' perspective informed their practice in conducting a clinical assessment:

I think I complete the assessment all the time with patient's consent no matter how I feel, so I can offer an accurate description/diagnosis of what is happening. By doing this, I help in ensuring that the patients are presented with the most accurate information to help guide them come with an informed decision regarding their healthcare decisions. (P17, B7)

Some participants described selecting the most appropriate approach to assessment which is tailored based on the patient's clinical status:

Perform an assessment the type of which depends on how the patient is presenting. I.e a‐e, drabc, ecg etc. (P30, B8)

##### 5.2.1.3 The Stress Nurses Feel and Its Perceived Impact

Most found assessment stressful.

Adrenaline kicks in. (P1, B6)

It was commonly believed that the stress participants experienced increased their resolve to be more thorough and systematic in conducting their assessment, and therefore benefitted not hindered their practice.

I stay calm and focus on patient's needs and make sure nothing missed from the diagnosis and treatment… Panic kills both ways. (P32, B5)

##### 5.2.1.4 Temporal Dimensions of Nurses' Assessments

Responses also conveyed that assessment is not an isolated event, rather that it is situated in time, and spans the past, present and future. Factors from the past included patient history and physiological trends.

…which may be due to his medical history and … other underlying problems…. (P16, B5)

…Track changes over time. (P31, B6)

Responses orientated in the present described the importance of urgency.

I am therefore always undertaking quick A‐Es certainly when first meeting patients. (P5, B5)

I feel like I need to be proactive and get this sorted asap. (P28, B7)

Pre‐emptive responses encompassed anticipating patient deterioration and repeating patient assessment or instigating treatments proactively.

…you get to know the signs they exhibit that they are deteriorating, and you can foresee the task and interventions that will be needed. (P6, B5)

#### 5.2.2 Theme Within the Education and Experience Domain

##### 5.2.2.1 Nurses' Belief in the Importance of Education and Experience

Participants who felt confident often proffered education and experience as justifications for feeling so.

I feel confident in assessing deteriorating patients as I have over 20 years' experience in CCU nursing… I feel the scope of my patient assessments is driven by experience…. (P33, B7)

I have also completed a degree in cardiology and finished advanced assessment modules both in full physical assessment and focused cardio/respiratory assessment. All of which are helpful in my confidence level. (P17, B7)

Participants suggestions for improvement often centred around educational strategies highlighting the perceived importance of education.

Nurse educators going through specific common scenarios in the wet lab situation to run through how to assess, treat and manage these patients. (P31, B6)

### 5.3 Quantitative Results

**FIGURE 3:**
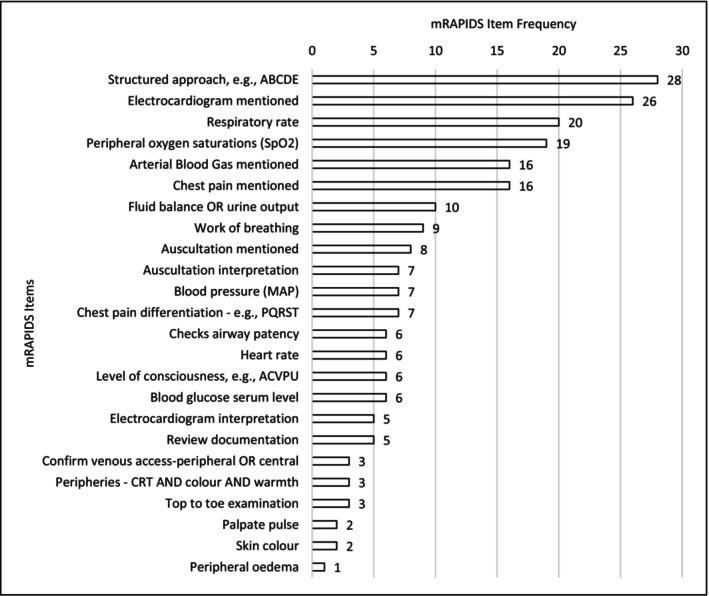
mRAPIDS item frequency. ABCDE, airway, breathing, circulation, disability and exposure assessment framework; ACVPU, alert, new confusion, voice, pain and unresponsive; CRT, capillary refill time; PQRST, provocation/palliation, quality, region/radiation, severity and timing.

The median mRAPIDS score of participants' assessments was 5 (IQR 4–8) out of 24 (range 2–16), with ‘structured approach’ (e.g., ABCDE) the most mentioned item (Figure 3). Having assessed the patient, 2 participants formed correct (6%), 11 partially correct (32%) and 21 incorrect (62%) impressions about the patient's condition. Of the suggested responses, 28 (82%) were correct, and 6 (18%) incorrect.

The only statistically significant difference in median mRAPIDS scores was between participants who formed correct and incorrect patient impressions (*p* = 0.015), but not with the partially correct group (Table 4). Although the correct group had a relatively narrow range of mRAPIDS scores (13–14), certainty was limited by the small group size (*n* = 2). Combined correct answers (partially correct and correct) (*n* = 13 Md = 7) versus incorrect (*n* = 21 Md = 5) were significantly different (*p* = 0.035). Although, combining correct groups improved group size, it was difficult to justify because of the different criteria for a partially correct (myocardial infarction OR pulmonary oedema OR heart failure) versus correct answers (myocardial infarction AND pulmonary oedema OR heart failure). There was no association between participants' impression of the patient and their suggested clinical response (Fisher–Freeman–Halton exact test, *p* = 0.755).

Of the mRAPIDS tool items, only ‘review documentation’ was directly relevant to the temporal theme. The five participants who mentioned ‘review documentation’ had a higher median mRAPIDS score (*p* = 0.002). Despite this, their impression was not significantly different (Fisher–Freeman–Halton exact test, *p* = 0.501), nor was the suggested response (Fisher's exact test, *p* = 0.559). The mRAPIDS tool did not quantitatively capture the other aspects within the temporal theme. Quantitative subgroup analysis of the participants who mentioned the other temporal aspects showed that their mRAPIDS score, impression and response were not significantly different. Median mRAPIDS scores across demographic categories (including education and experience) did not differ significantly.

### 5.4 Mixed Methods Results

#### 5.4.1 The Act of Assessment Domain Results Integration

Integrating quantitative and qualitative findings through comparison of how they ‘fit’ together showed overlap on the same central phenomenon (the act of assessment), and broader nonoverlapping viewpoints (Younas and Durante 2022). Despite most participants mentioning the use of a structured approach, quantitatively measured scope was narrow and patients' impressions mostly incorrect. These quantitative findings can be further understood through participants' qualitatively derived perceptions—that NEWS2 may not promote adequate assessment, the role of tailored scope and the influence of stress on assessment. Qualitative findings also richly conveyed temporal dimensions that were quantitatively less well captured.

#### 5.4.2 Education and Experience Domain Results Integration

Although qualitative findings suggested participants held education and experience in high regard, in quantitative analysis scope of assessment did not differ significantly with education or experience. In this domain, the ‘fit’ between quantitative and qualitative findings was therefore conflicting (disconcordant).

## 6 Discussion and Conclusion

The use of a convergent mixed methods design enabled identification of two themes. The *act of assessment* domain revealed that scope of assessment was low, which may have been influenced by tailoring, NEWS2, stress and temporal dimensions. In the *education and experience* domain, neither education nor experience seemed to affect scope of assessment despite both being emphasised by participants.

The scope of our participants' assessments was measured with the mRAPIDS tool that was developed from Liaw et al. (2011) RAPIDS tool. RAPIDS scores in the original study were also low but could not be meaningfully compared to ours because participants were nursing students, and it included more items (*n* = 31) that extended beyond just assessment. The involvement of researchers with subject matter expertise and clinical stakeholders in the development and validation of our mRAPIDS tool increased the likelihood that we had accurately measured the scope of our participants' responses.

Some of our participant's responses illustrated the importance of clinical assessment occurring across time where patient history and previously recorded vital signs shape how an assessment is conducted and prompt consideration of interventions that may be required in the future. In our work, this was represented by the temporal dimensions theme. Temporal dimensions expressed by participants and captured in the qualitative data were not well reflected in the mRAPIDS scores. In the quantitative analysis of participant's whose answers featured temporal dimensions, the scope of their assessments was not broader (aside from those who reviewed documentation), nor were the impressions they formed or responses they suggested more accurate. This implies that a nurse's awareness of the temporal dimensions has no bearing on the scope of their assessment, nor the accuracy of the clinical impression formed. As our results reflect ‘work‐as‐reported’ (nurses describing their own practice), which often differs from work‐as‐done (the actual work performed) (Salvendy and Karwowski 2021) our certainty in this argument is limited and further practice‐based research would be needed to explore this.

It is long‐established that most of the information required to reach an accurate diagnosis is obtained from patient history (Roshan and Rao 2000), and therefore, temporal dimensions should feature in patient assessment. The mRAPIDS tool assesses scope of assessment at a single time point with no recognition of the need to consider previous information or consider changes over time (e.g., trends in vital signs) raising questions about its validity.

In other acute settings, the scope of nurse and midwife assessments has also been found to be low, with assessment tending to focus on vital signs, not broader signs and symptoms (Osborne et al. 2015). In our study, whilst the monitoring of some vital signs (respiratory rate, peripheral oxygen saturations) was mentioned frequently, other vital signs (heart rate, level of consciousness) were given lower status. This may have been an unintended consequence of how the vignette was presented. As all vital signs were displayed participants may have felt they did not need to be repeated which could have lowered their mRAPIDS score. An alternative explanation is that there are specific barriers to nurses completing a complete set of vital signs every time they assess a patient. This is consistent with findings from other research where nursing staff were observed performing partial monitoring; that is, monitoring some vital signs but not others (Smith et al. 2020).

Integration of quantitative and qualitative findings provided potential explanations for participants' low mRAPIDS scores. Similar to our NEWS2 theme, other research examining NEWS has revealed senior nurses have concerns about it encouraging narrow task‐based patient assessment for more junior nurses (Spångfors et al. 2019; Smith et al. 2020). In cardiac specialities, NEWS2 has demonstrated low predictive accuracy of ICU admission, cardiac arrest and medical emergencies (Alhmoud et al. 2023). Junior nurses' reliance on an imperfect tool may signal a knowledge gap and highlights the importance of an appropriate skill mix in CCUs to ensure novice practitioners receive adequate support from senior colleagues. As well as support from peers of the same profession, findings from a recent systematic review highlight the importance of interprofessional collaboration too (Hong et al. 2023).

The tailoring theme demonstrated how the scope of assessment might be intentionally shaped. Participants described engagement in deliberate tailored assessment that was safe and empathetic. These findings highlighted the complexity of the assessment of the nurses' role in the afferent limb of the RRS. This suggests that conceptually depicting nurses' assessments as linear discrete processes, that is, the ABCDE framework, is an oversimplification. Our approach to quantitative measurement of scope with the mRAPIDS tool was framed by the ABCDE approach, which therefore also prompts questions about the validity of the tool. Although others have concluded that deviation from ABCDE frameworks reflects insufficient knowledge (Schoeber et al. 2022), there is arguably a need to better understand nurses' assessment in practice to inform the development of frameworks that permit more tailored approaches to clinical assessments.

The stress many participants described may have influenced the scope of their assessment. Research has tended to focus on the stress that nurses feel when escalating patient deterioration (Massey, Chaboyer, and Anderson 2017). More recent research in the acute hospital ward setting has focussed on stress during assessment and shown an even divide amongst participants who felt that stress was a barrier or enabler (Smith et al. 2021). Most of our participants believed stress enhanced their performance. Besides clinical areas, an important difference between our study and Smith et al.'s (2021) was their inclusion of healthcare assistants who may have different emotional responses to patient deterioration. Exploration of the role of health care assistants was beyond the remit of our study but warrants empirical exploration in specialist clinical areas (e.g., CCUs).

Having assessed the deteriorating CCU patient depicted in the vignette, participants were then asked for their clinical impression (clinical conclusion). This required clinical judgement—a reflective and reasoning process drawing on available data (Connor et al. 2022). Although guidelines and current policy acknowledge the importance of exercising clinical judgement, they offer no detail on how this might be achieved (Royal College of Physicians 2017), despite a substantial body of literature reporting clinical judgement theory (Thirsk et al. 2022).

This study showed a moderate signal that the assessments from participants who arrived at correct impressions had greater scope than those who formed incorrect impressions. The assessments of the two participants in this study that formed correct impressions had significantly greater scope (13 and 14 items) than incorrect impressions. Also, the median scope of assessment of combined correct and partially correct groups (*n* = 13) was greater than the incorrect (7 vs. 5). For the intentionally ambiguous scenario presented to our participants, it is plausible that the collection and interpretation of more clinical data improved the accuracy of their impression, which would align with current theoretical understanding of clinical judgement (Thirsk et al. 2022).

Within the broader context of the RRS, it could be argued that it does not matter whether a nurse forms a correct patient impression or not. Such an argument could be advanced from the absence of a significant association between the patient impression our participants arrived at and the likelihood of them suggesting a correct or incorrect response. However, our criteria for correct responses were limited to monitoring and escalation. As the tailoring theme in our work illustrates, the response to patient deterioration is much more complex and likely contingent on nurses' forming a correct impression of the patient's condition. Recent research employing human factors modelling has similarly demonstrated the complexity of nurses' responses (Ede et al. 2024). Our participants' suggested response was therefore arguably too crude a proxy to quantify the clinical utility of bedside nurses' reaching correct clinical conclusions.

When confronted by patient deterioration, nurses often have very little time for patient assessment and clinical judgement (Al‐Moteri et al. 2020). In this study, despite the absence of time restrictions, participants may have defaulted to how they approach assessment in their day‐to‐day practice, which may explain the often‐narrow scope of assessments and few correct impressions.

For our participants, greater experience was not associated with broader assessments. It is known that those with greater experience have a greater predilection for using pattern recognition that draws on less assessment data (Connor et al. 2022). Our results reflect that even with experience, assessment is fallible to bias and error, which re‐iterated the importance of clinical judgement. Level of education was also not associated with broader assessments. Greater education is known to be an antecedent to better clinical judgement (Connor et al. 2022). Yet, as with experience, greater capacity for pattern recognition may have confounded any difference. Evidently, pattern recognition offers some explanation of why our results differ from other studies.

Lack of statistical power and methodological limitations of our study aside, there may also be room to improve the teaching of clinical judgement. Current undergraduate education tends to produce nurses who are unprepared to exercise clinical judgement—a shortfall potentially exacerbated by the impact of COVID‐19 on university education (Levett‐Jones et al. 2010; McGarity et al. 2023).

### 6.1 Strengths and Limitations

A strength of this research was the development and validation of the mRAPIDS tool to measure the scope of nurses' assessments of deteriorating CCU patients, although this study has highlighted the validity of the mRAPIDS tool could be improved further. Another strength was the contribution of the study‐specific clinician and patient stakeholder group.

Several limitations need to be acknowledged, the first being sample size. In this type of mixed methods research, the same participants provide qualitative and quantitative data, and therefore, a balance was struck between producing manageable amounts of qualitative data and ensuring sufficient quantitative statistical power. The brevity of some responses meant data was often ‘thin’ (i.e., insufficiently rich), which had to be offset (to an extent) by increasing the sample size.

Non‐response and attrition bias were likely further limitations. Due to social media recruitment, it was not possible to calculate an exact response rate, but it was likely low. The completion rate was also low, which may have been because of the response burden (time and effort) from the numerous open questions.

Methodological limitations meant that understanding nurses' assessments was reliant upon participants own descriptions of it (work‐as‐disclosed). It was hoped that the steps taken to preserve confidentiality and develop a realistic vignette would reduce the gap between work‐as‐disclosed and work‐as‐done. However, these steps could not have overcome the limited ecological validity inherent in vignette‐based studies.

### 6.2 Implications

This inductive study provides some preliminary insights into understanding nurses' assessments of deteriorating CCU patients.

Although the A‐E assessment is a theoretically reasonable framework, our findings suggest that it is not used as intended. We suggest that there is instead a need for further research to better understand nurses' ‘real‐world’ assessments to inform the development of an alternative framework that maintains a safe priority‐driven approach to clinical assessment but simultaneously enabling more clinical judgement.

This study focussed on nurses, but it is well established that healthcare assistants are important actors within the afferent limb. Current shortfalls of nurses increase the likelihood that healthcare assistants will remain central actors, even in specialist settings (e.g., CCUs). In the United Kingdom, it is also possible that the recently introduced nursing associate role may occupy a role in the afferent limb, although this has yet to be empirically examined. Further research with healthcare assistants and nursing associates would provide a more complete understanding of assessment of deteriorating patients in CCUs.

Within this study, the importance of clinical judgement became evident. There is room in current clinical guidelines and policy for more detailed recommendations on how to perform and teach clinical judgement. Theory‐driven research examining the interface between RRSs and clinical judgement could inform such recommendations. Dual process theory is a widely accepted theory of clinical judgement derived from cognitive and social psychology empirical evidence that could offer an appropriate platform from which to conduct further research (Thirsk et al. 2022).

### 6.3 Conclusion

In this mixed‐method study, the scope of nurses' assessments of deteriorating CCU patients was explored. Primary findings were generated from integrating qualitative and quantitative data in two main domains. In the *act of assessment* domain, the measured scope of participants' assessments was low, which could be further understood with themes derived from nurses concurrently collected perceptions around NEWS2, tailoring, stress and temporal dimensions. For the *education and experience* domain, nurses perceived both to be important, but neither made a difference to the scope of assessment. Participants' assessments generally did not demonstrate complete ABCDE assessment, which is widely accepted as best practice. Implications spanning policy, education and practice are suggested.

## Author Contributions

**Nicholas Woolfe Loftus:** conceptualisation, formal analysis, funding acquisition, investigation, methodology, writing original draft. **Duncan Smith:** conceptualisation, funding acquisition, methodology, supervision, writing review and editing. **Leanne M Aitken:** conceptualisation, funding acquisition, methodology, supervision, writing review and editing.

## Conflicts of Interest

The authors declare no conflicts of interest.

## Statistics

The statistics were checked prior to submission by statistician Saiful Islam. His email address is as follows: saiful.islam@city.ac.uk.

## Supporting information


Appendix S1

Appendix S2

## Data Availability

The data that support the findings of this study are available on request from the corresponding author. The data are not publicly available due to privacy or ethical restrictions.
