# Supplementary material for: The Scope of Nurses' Assessment of Deteriorating Patients in Coronary Care Units: A Mixed Methods Study
Source: J Clin Nurs. 2025 Jan 24;34(4):1250–63. doi: 10.1111/jocn.17500 (PMC11933513; doi:10.1111/jocn.17500)
Supplement: Supplementary file 1 — Appendix S1 [file JOCN-34-1250-s001.pdf]

JARS–**Mixed** | Table 1

Mixed Methods Article Reporting Standards (MMARS)
Information Recommended for Inclusion in Manuscripts That Report the Collection and Integration of Qualitative and Quantitative Data

Title Page

Title

- See the JARS–Qual and JARS–Quant Standards.

Guidance for Authors

- Refrain from using words that are either qualitative (e.g., "explore," "understand") or quantitative (e.g., "determinants," "correlates"), because mixed methods stands in the middle between qualitative and quantitative research.
- Reference the mixed methods, qualitative methods, and quantitative methods used.

Author Note

- See the JARS–Qual and JARS–Quant Standards.

Abstract

- See the JARS–Qual and JARS–Quant Standards.
- Indicate the mixed methods design, including types of participants or data sources, analytic strategy, main results/findings, and major implications/significance.

Guidance for Authors

- Specify the type of mixed methods design used. See the note on types of designs in the Research Design Overview section of this table.
- Consider using one keyword that describes the type of mixed methods design and one that describes the problem addressed.
- Describe your approach(es) to inquiry and, if relevant, how intersecting approaches to inquiry are combined when this description will facilitate the review process and intelligibility of your paper. If your work is not grounded in a specific approach(es) to inquiry or your approach would be too complicated to explain in the allotted word count, however, it would not be advisable to provide explication on this point in the abstract.

Introduction

Description of Research Problems/Questions

- See the JARS–Qual and JARS–Quant Standards.

Description of Research Problems/Questions *(continued)*

Guidance for Authors

- This section may convey barriers in the literature that suggest a need for both qualitative and quantitative data.

Guidance for Reviewers

- Theory or conceptual framework use in mixed methods varies depending on the specific mixed methods design or procedures used. Theory may be used inductively or deductively (or both) in mixed methods research.

Study Objectives/Aims/Research Goals

- See the JARS–Qual and JARS–Quant Standards.
- State three types of research objectives/aims/goals: qualitative, quantitative, and mixed methods. Order these goals to reflect the type of mixed methods design used.
- Describe the ways approaches to inquiry were combined, as it illuminates the objectives and mixed methods rationale (e.g., descriptive, interpretive, feminist, psychoanalytic, postpositivist, critical, postmodern, constructivist, or pragmatic approaches).

Guidance for Reviewers

- A mixed methods objective, aim, or goal may not be familiar to reviewers. It describes the results to be obtained from using the mixed methods design type where “mixing” or integration occurs (e.g., the aim is to explain quantitative survey results with qualitative interviews in an explanatory sequential design). For instance, the goal of a qualitative phase could be the development of a conceptual model, the goal of a quantitative phase could be hypothesis testing based upon that model, and the goal of the mixed methods phase could be to generate integrated support for a theory based upon quantitative and qualitative evidence.

Method

Research Design Overview

- See the JARS–Qual and JARS–Quant Standards.
- Explain why mixed methods research is appropriate as a methodology given the paper’s goals.
- Identify the type of mixed methods design used and define it.
- Indicate the qualitative approach to inquiry and the quantitative design used within the mixed methods design type (e.g., ethnography, randomized experiment).

© 2020 American Psychological Association

Research Design Overview *(continued)*

- If multiple approaches to inquiry were combined, describe how this was done and provide a rationale (e.g., descriptive, interpretive, feminist, psychoanalytic, postpositivist, critical, postmodern, constructivist, or pragmatic approaches), as it is illuminating for the mixed method in use.
- Provide a rationale or justification for the need to collect both qualitative and quantitative data and the added value of integrating the results (findings) from the two databases.

  Guidance for Reviewers

  - Because mixed methods research is a relatively new methodology, it is helpful to provide a definition of it from a major reference in the field.
  - Mixed methods research involves rigorous methods, both qualitative and quantitative. Refer to the JARS–Qual standards (qualitative) and JARS–Quant standards (quantitative) for details of rigor.
  - One of the most widely discussed topics in the mixed methods literature would be research designs. There is not a generic mixed methods design but rather multiple types of designs. Basic, core designs include convergent design, explanatory sequential design, and exploratory sequential design. Although the names and types of designs may differ among mixed methods writers, a common understanding is that procedures for conducting a mixed methods study may differ from one project to another. Further, these basic procedures can be expanded by linking mixed methods to other designs (e.g., an intervention or experimental trial mixed methods study), to theories or standpoints (e.g., a feminist mixed methods study), or to other methodologies (e.g., a participatory action research mixed methods study).

Participants or Other Data Sources

- See the JARS–Qual and JARS–Quant Standards.
- When data are collected from multiple sources, clearly identify the sources of qualitative and quantitative data (e.g., participants, text), their characteristics, and the relationship between the data sets, if there is one (e.g., an embedded design).
- State the data sources in the order of procedures used in the design type (e.g., qualitative sources first in an exploratory sequential design followed by quantitative sources), if a sequenced design is used in the mixed methods study.

  Guidance for Authors

  - Because multiple sources of data are collected, separate descriptions of samples are needed when they differ. A table of qualitative sources and quantitative sources is helpful. This table could include type of data, when data were collected, and from whom. This table might also include study aims/research questions for each data source and anticipated outcomes of the study. In mixed methods research, this table is often called an "implementation matrix."
  - Rather than describe data as represented in numbers versus words, it is better to describe sources of data as open-ended information (e.g., qualitative interviews) and closed-ended information (e.g., quantitative instruments).

Researcher Description

- See the JARS–Qual Standards.

  Guidance for Authors

  - Because mixed methods research includes qualitative research, and reflexivity is often included in qualitative research, we recommend statements as to how the researchers' backgrounds influence the research.

  Guidance for Reviewers

  - It is helpful to establish in a publication the researchers' experiences (or research teams' experiences) with both qualitative and quantitative research as a prerequisite for conducting mixed methods research.

Participant Recruitment

Participant Sampling or Selection

- See the JARS–Qual and JARS–Quant Standards.
- Describe the qualitative and the quantitative sampling in separate sections.
- Relate the order of the sections to the procedures used in the mixed methods design type.

Participant Recruitment

- See the JARS–Qual and JARS–Quant Standards.
- Discuss the recruitment strategy for qualitative and quantitative research separately.

Data Collection

Data Collection/Identification Procedures

- See the JARS–Qual and JARS–Quant Standards.

Recording and Transforming the Data

- See the JARS–Qual Standards.

Data Analysis

- See the JARS–Qual and JARS–Quant Standards.
- Devote separate sections to the qualitative data analysis, the quantitative data analysis, and the mixed methods analysis. This mixed methods analysis consists of ways that the quantitative and qualitative results will be "mixed" or integrated according to the type of mixed methods design used (e.g., merged in a convergent design, connected in explanatory sequential designs and in exploratory sequential designs).

Validity, Reliability, and Methodological Integrity

- See the JARS–Qual and JARS–Quant Standards.
- Indicate methodological integrity, quantitative validity and reliability, and mixed methods validity or legitimacy. Further assessments of mixed methods integrity are also indicated to show the quality of the research process and the inferences drawn from the intersection of the quantitative and qualitative data.

© 2020 American Psychological Association

Findings/Results

Findings/Results Subsections

- See the JARS–Qual and JARS–Quant Standards.
- Indicate how the qualitative and quantitative results were "mixed" or integrated (e.g., discussion; tables of joint displays; graphs; data transformation in which one form of data is transformed to the other, such as qualitative text, codes, themes are transformed into quantitative counts or variables).

  Guidance for Authors

  - In mixed methods research, the Findings section typically includes sections on qualitative findings, quantitative results, and mixed methods results. This section should mirror the type of mixed methods design in terms of sequence (i.e., whether quantitative strand or qualitative strand comes first; if both are gathered at the same time, either qualitative findings or quantitative results could be presented first).

  Guidance for Reviewers

  - In mixed methods Results sections (or in the Discussion section to follow), authors are conveying their mixed methods analysis through "joint display" tables or graphs that array the qualitative results (e.g., themes) against the quantitative results (e.g., categorical or continuous data). This enables researchers to directly compare results or to see how results differ between the quantitative and qualitative strands.

Discussion

Discussion Subsections

- See the JARS–Qual and JARS–Quant Standards.

  Guidance for Authors

  - Typically, the Discussion section, like the Method and Findings/Results, mirrors in sequence the procedures used in the type of mixed methods design. It also reflects on the implications of the integrated findings from across the two methods.

© 2020 American Psychological Association
