# Supplementary material for: The Scope of Nurses' Assessment of Deteriorating Patients in Coronary Care Units: A Mixed Methods Study
Source: J Clin Nurs. 2025 Jan 24;34(4):1250–63. doi: 10.1111/jocn.17500 (PMC11933513; doi:10.1111/jocn.17500)
Supplement: Supplementary file 2 — Appendix S2 [file JOCN-34-1250-s002.docx]

**Supplementary Files**

Supplementary File 1: Recruitment Email


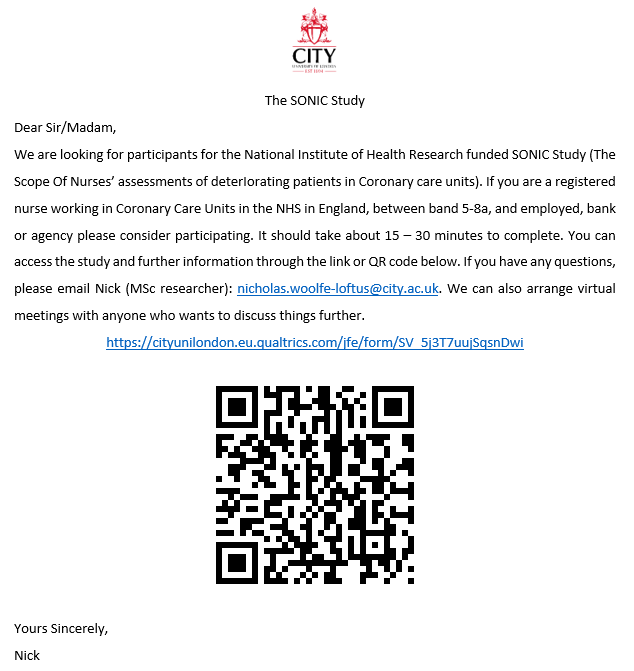


Supplementary File 2: Social media recruitment post


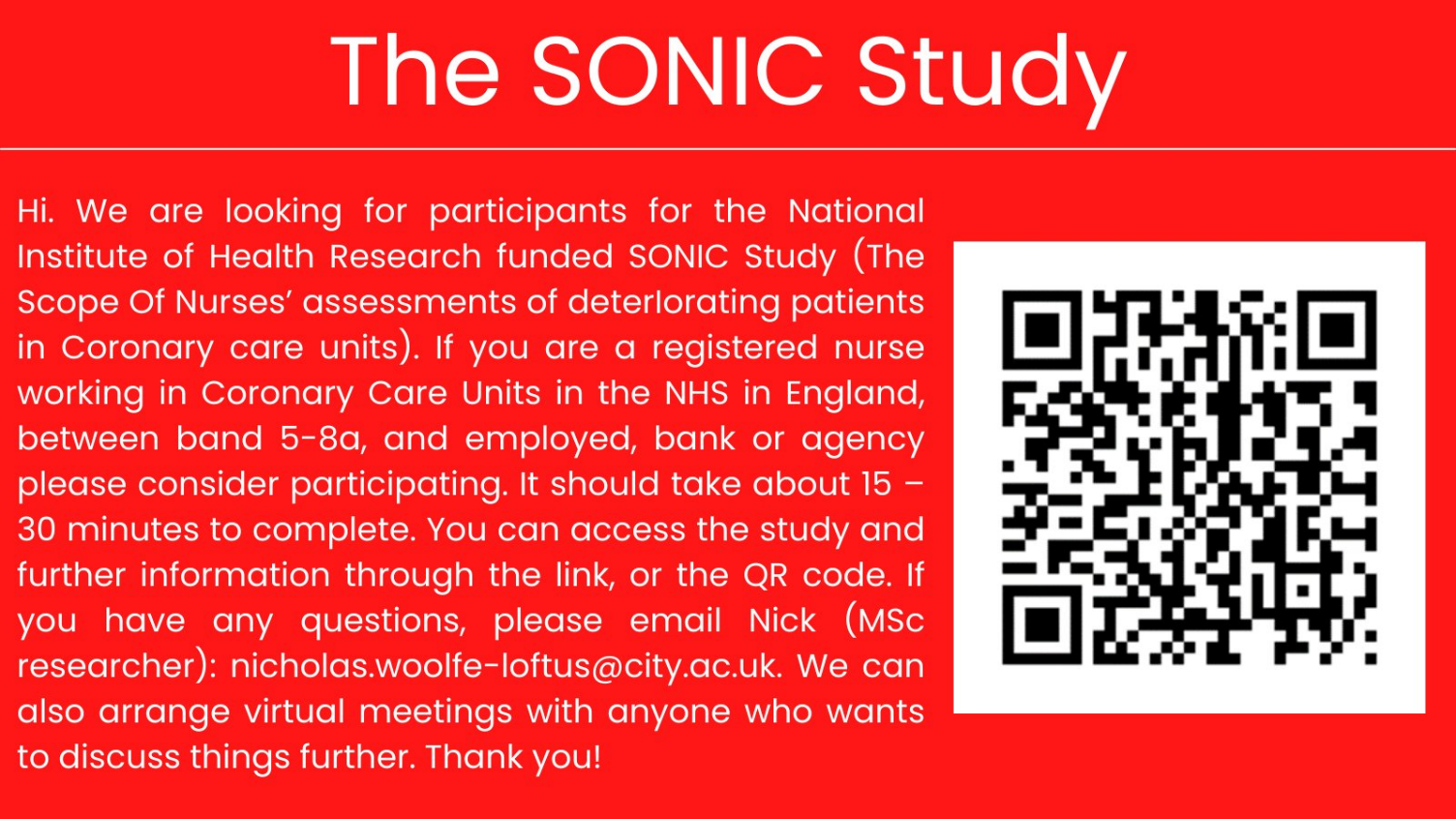


Supplementary File 3: Exported Qualtrics survey


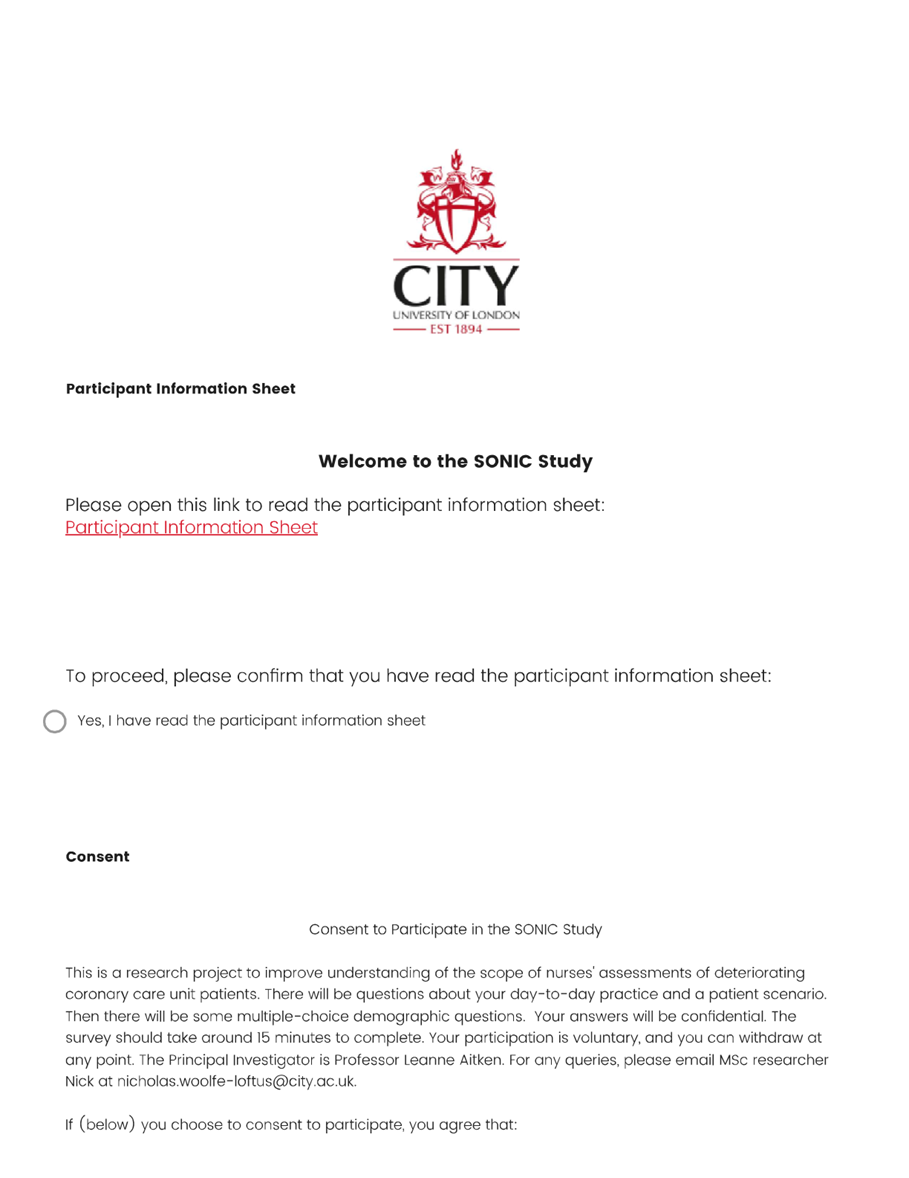

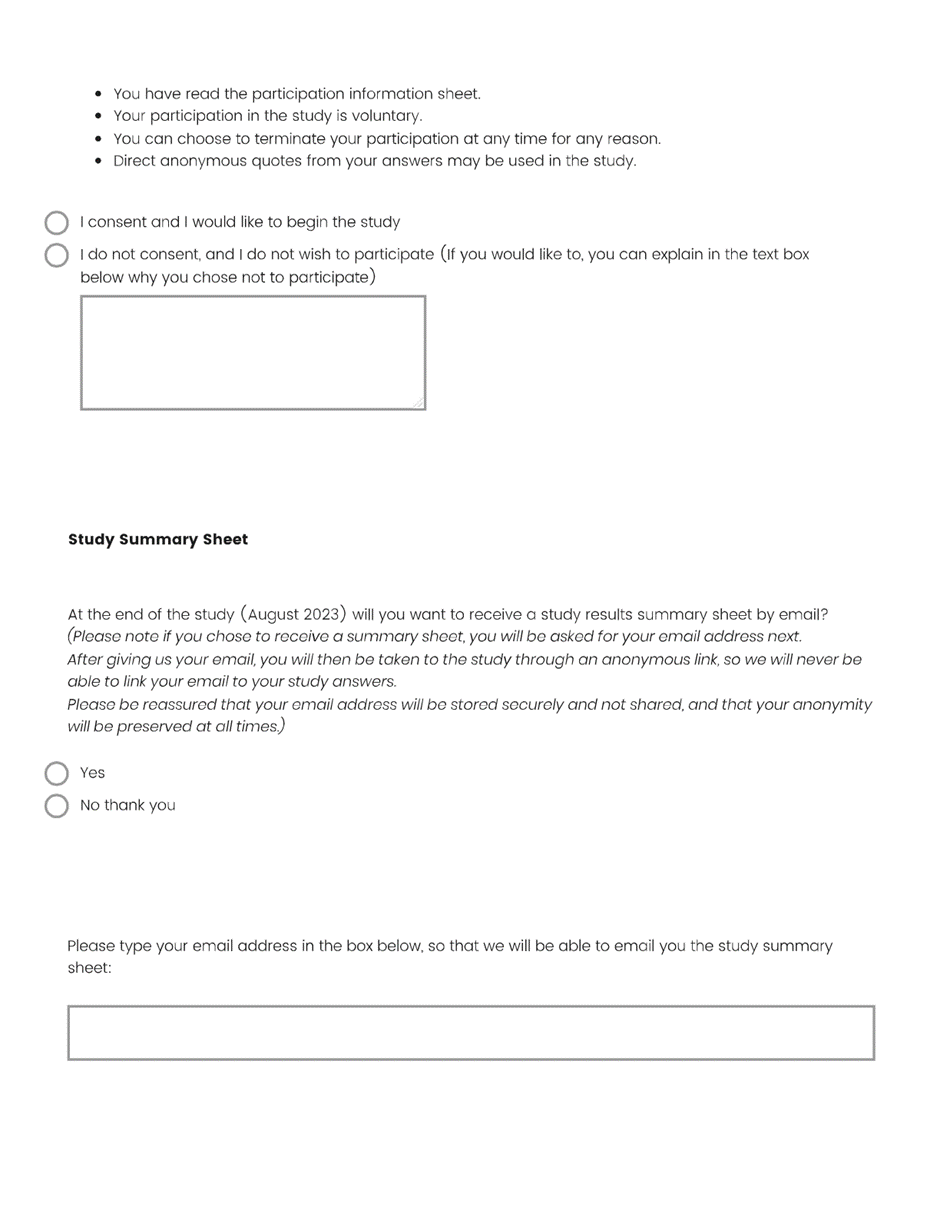


Supplementary File 3: Exported Qualtrics survey continued


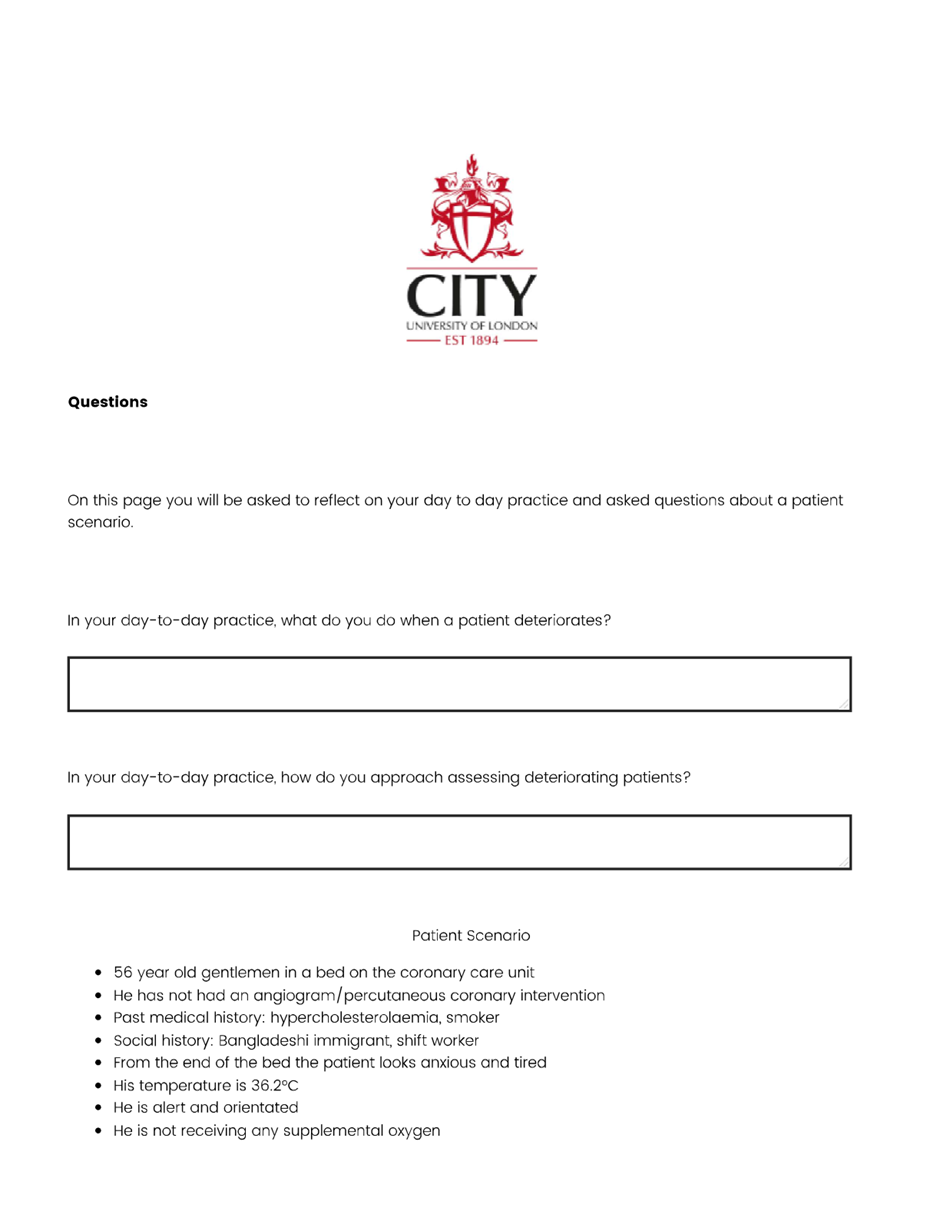

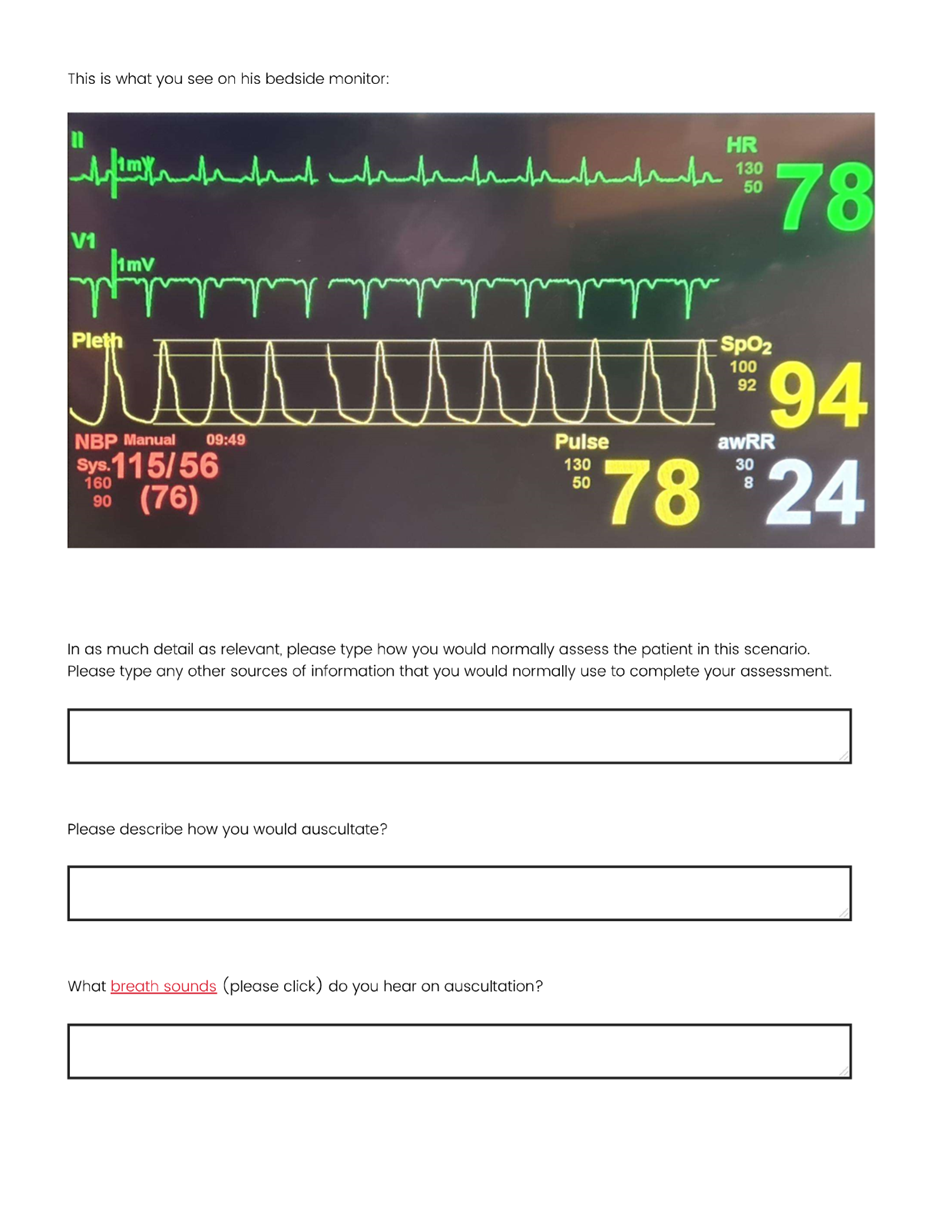


Supplementary File 3: Exported Qualtrics survey continued


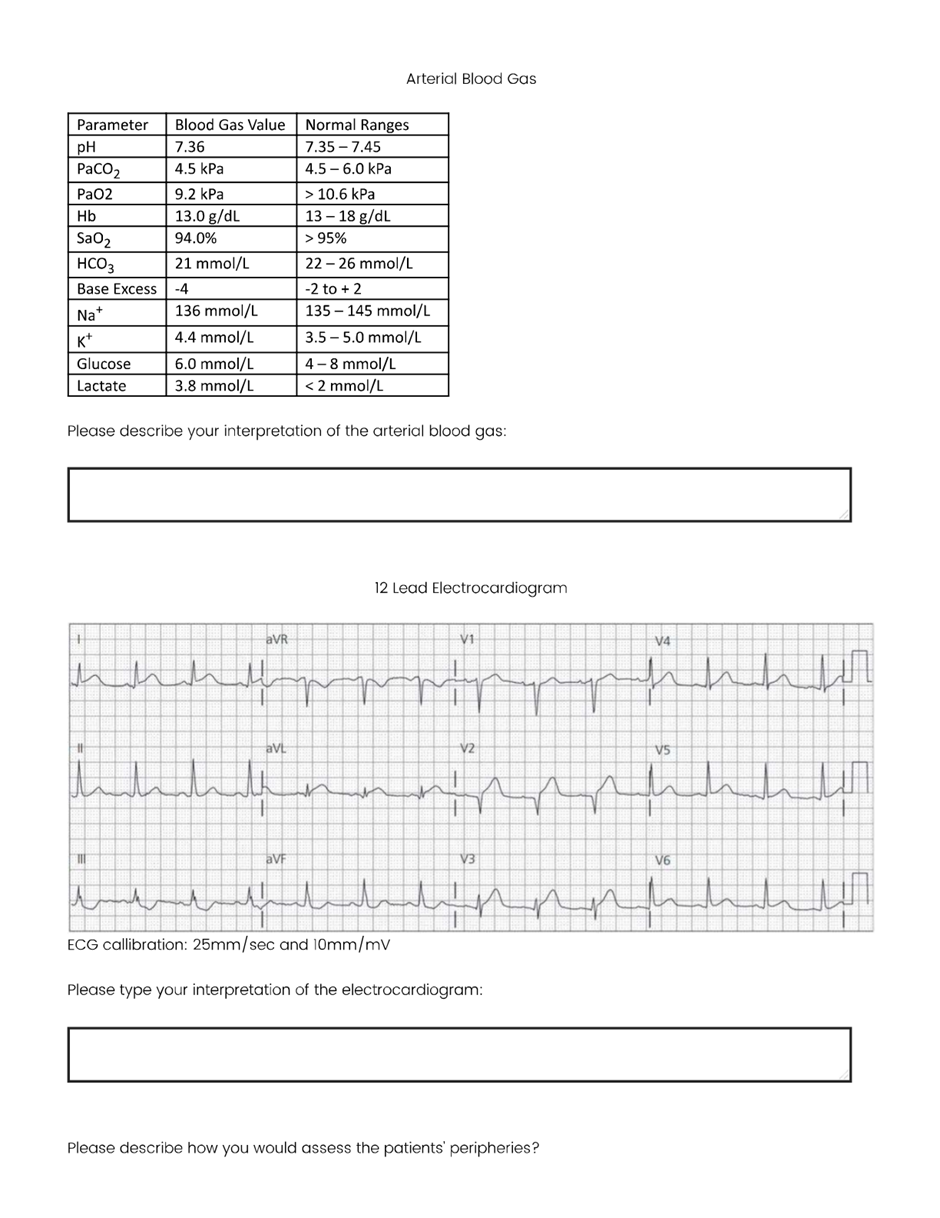

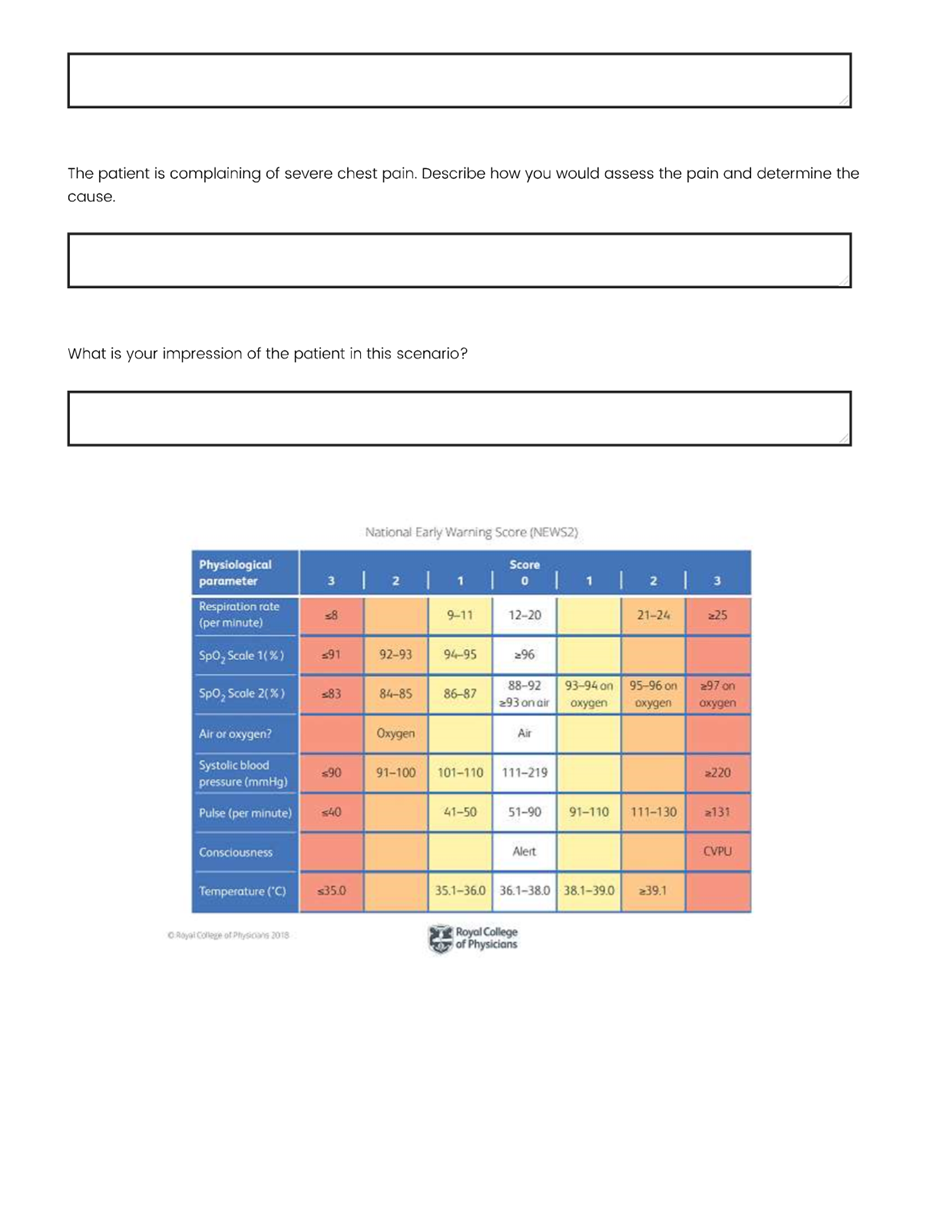


Supplementary File 3: Exported Qualtrics survey continued


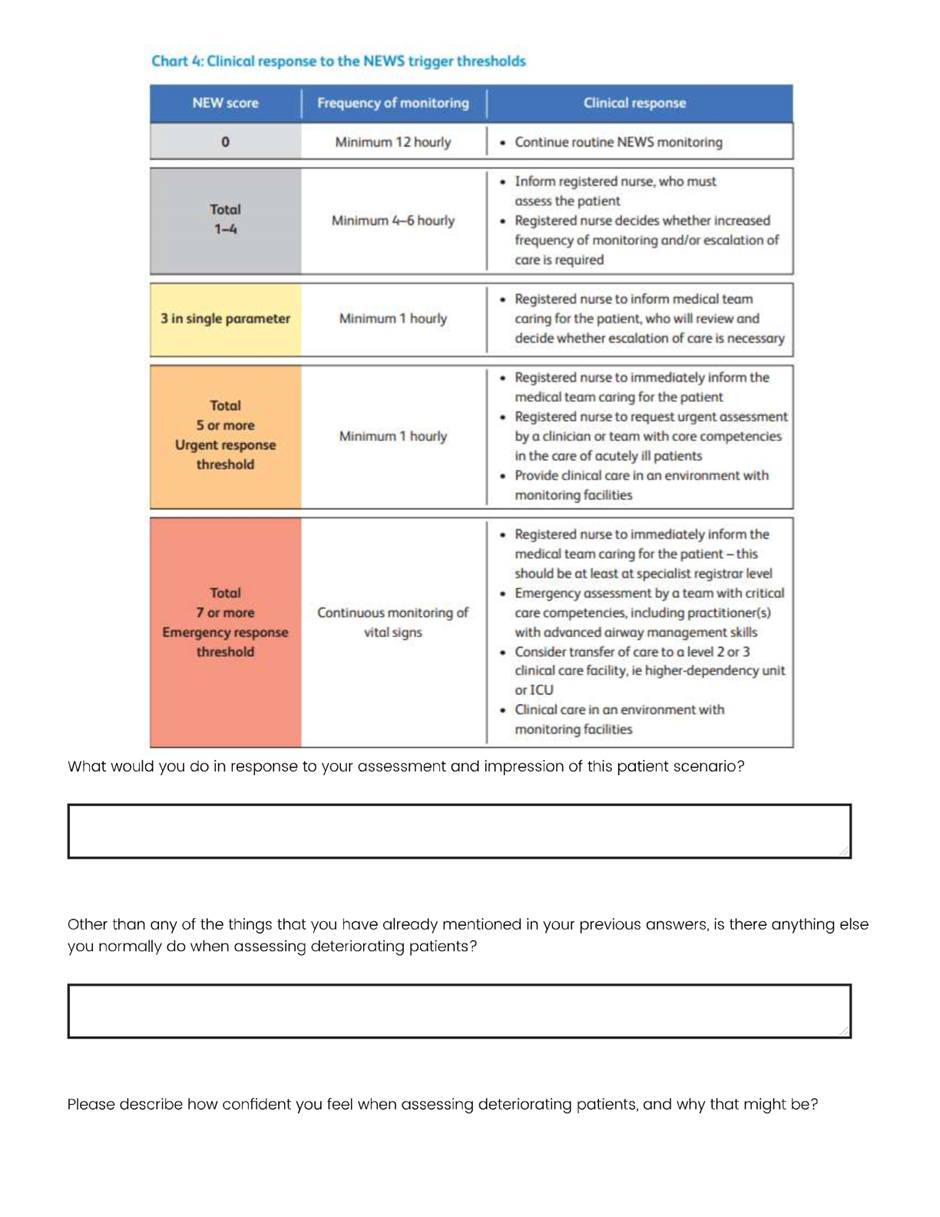

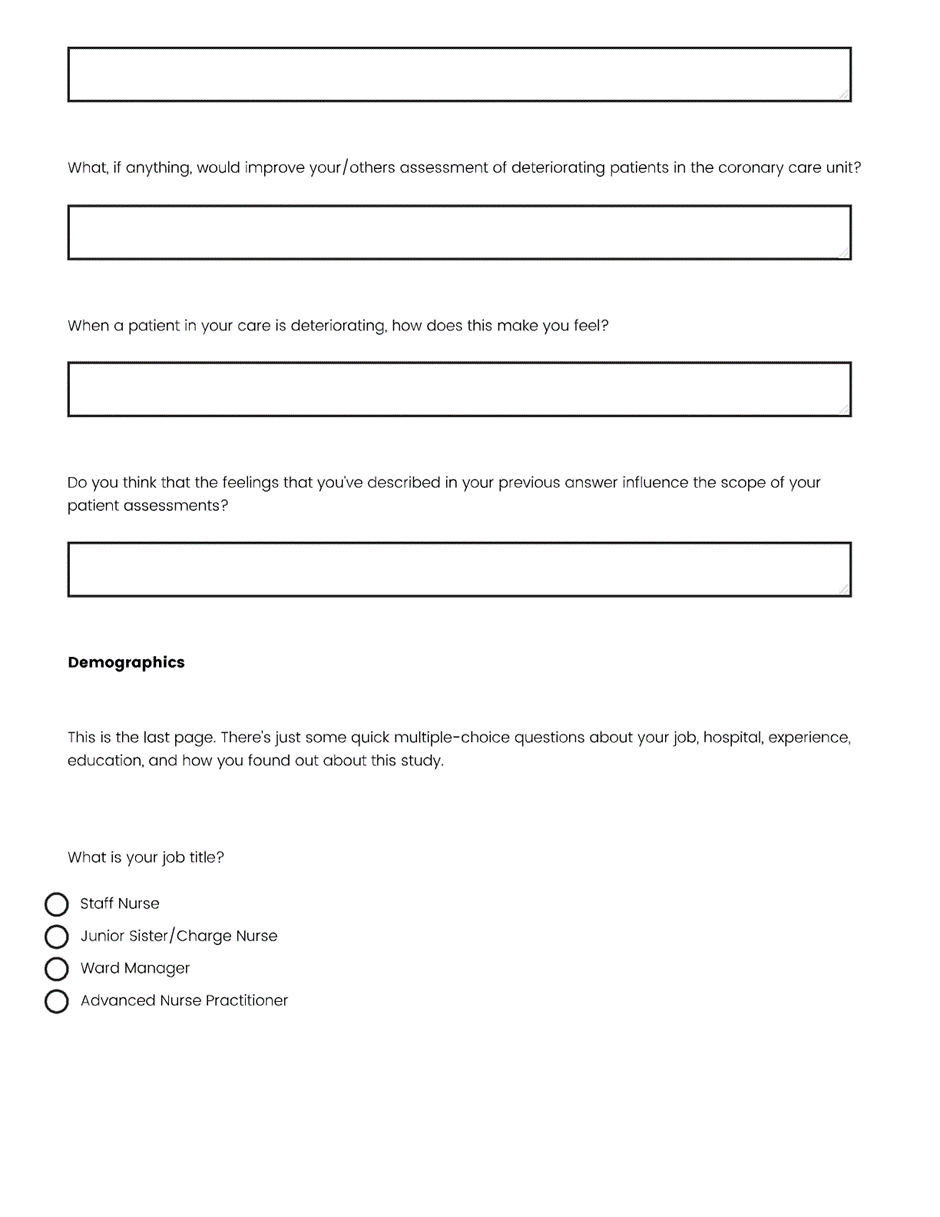


Supplementary File 3: Exported Qualtrics survey continued


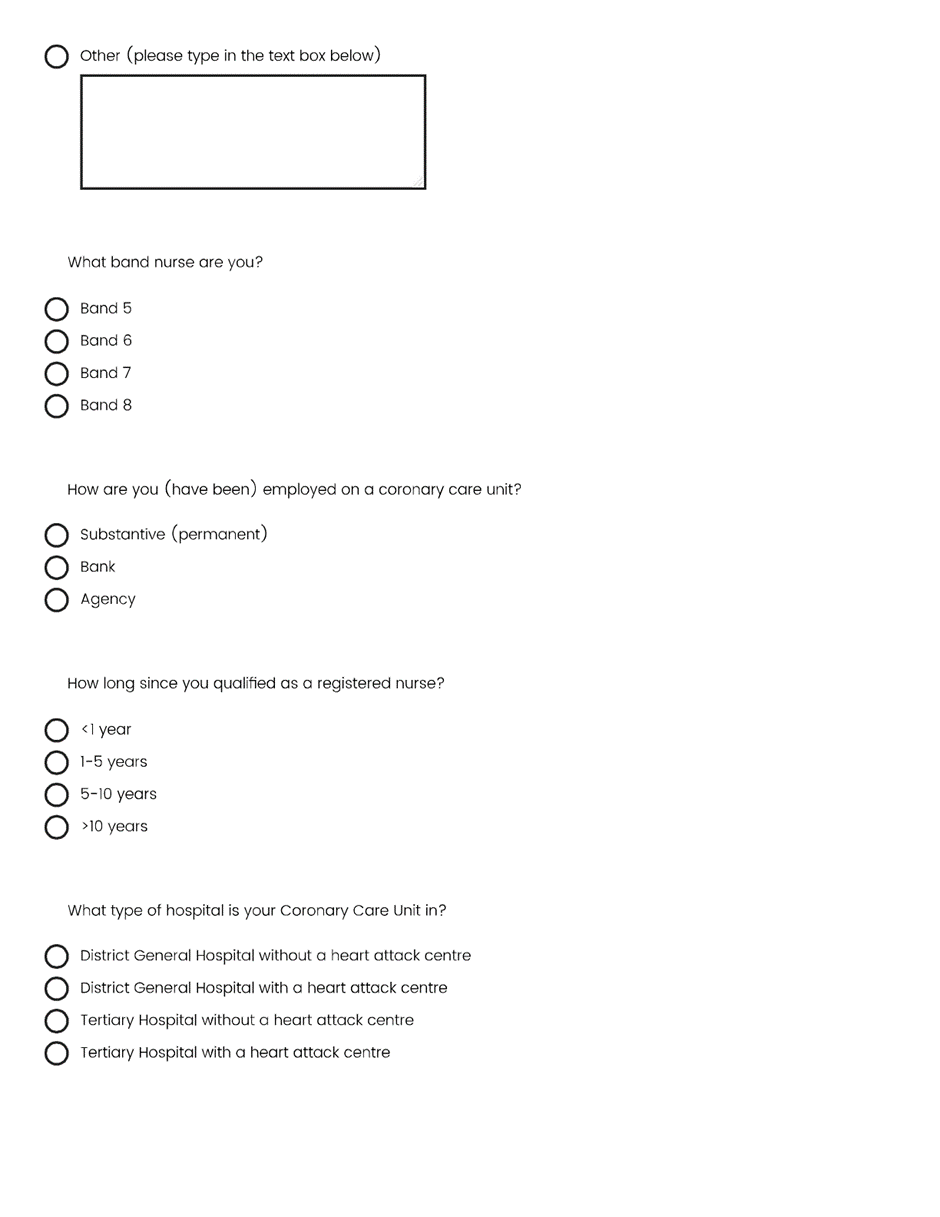

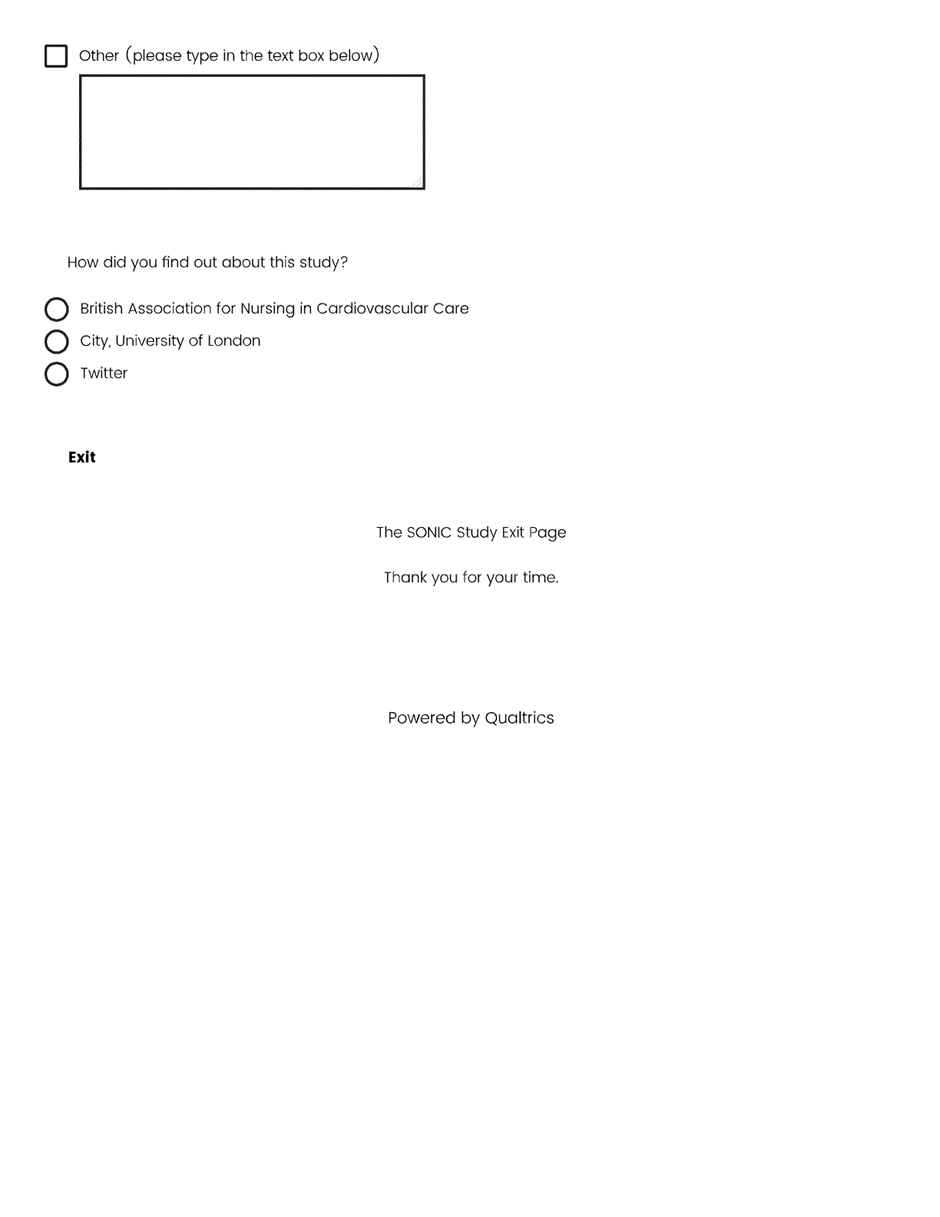


Supplementary File 4: Examples of pilot feedback and its impact

| Quote | Impact |
| --- | --- |
| ‘...some of the boxes allowed a lot of free text (which was visible to the reader), whereas others allowed text, but didn’t seem to want you to write much…’ | Boxes for free text entry standardised to an appropriate size. |
| Highlighted that a word was missing from this sentence: ‘You will be asked to reflect on your day-to-day practice. and asked a patient scenario’. | Reworded: ‘You will be asked to reflect on your day-to-day practice. and asked a patient scenario’. |
| ‘…being immediately confronted by the PIS (participant information sheet) makes the survey appear quite dense...’ | Participant information sheet instead incorporated as a link to click on. |
| ‘…normal convention is to place demographic questions at the end of a survey to avoid putting off participants by being asked straight away to disclose sensitive information…’ | Demographic questions moved from beginning to end of survey. |
| ‘…ECG calibration information not included…’ | ECG calibration information included. |

Supplementary File 5: mRAPIDS tool


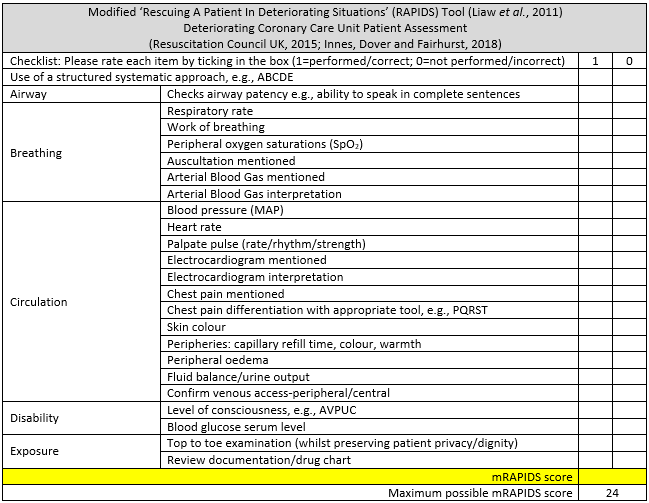


Supplementary File 6: Participant information sheet


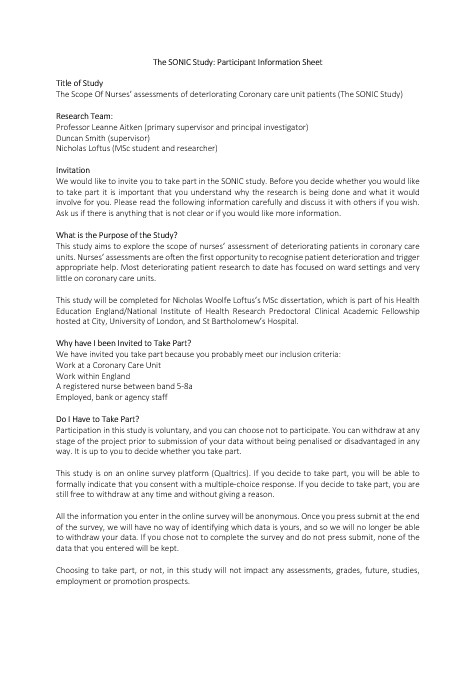

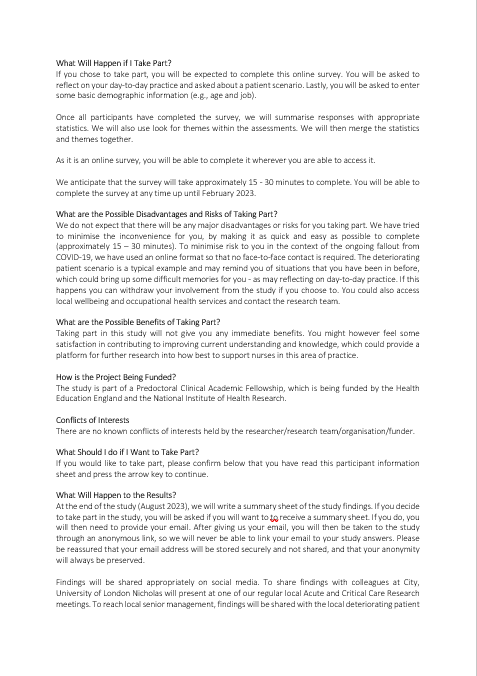


Supplementary File 6: Participant information sheet continued


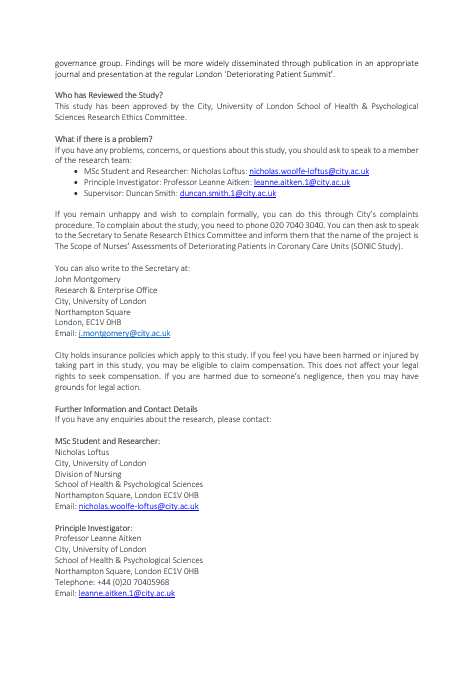

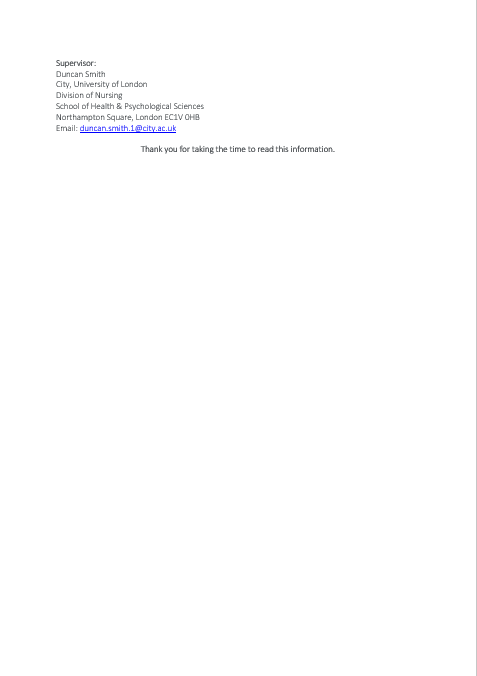


Supplementary File 7: Themes, codes, and quotes

| Themes | Codes | Data | Participant |
| --- | --- | --- | --- |
| 1. The stress nurses experience and its consequences | Traumatising legacy | ‘I still remember now a patient who died when I was a student nurse and a young patient a few years ago. Sometimes the memories are hard, but it also tells me I still care and I believe pushes me to continue’ | 9 |
|  |  | ‘Of course, when you know that there is a probability of a patient dieing due to sudden detioration it will affect you emotionally and psychological but we are training to look calm, professional and coordinated at those moments.’ | 16 |
|  | Caring for a deteriorating patient is stressful | ‘Adrenaline kicks in’ | 1 |
|  |  | ‘Nervous’ | 3 |
|  |  | ‘Anxious’ | 4 |
|  |  | ‘When I get a deteriorating patient, I get a bit anxious as I have to rely heavily on the team on duty with me.’ | 7 |
|  |  | ‘Nervous’ | 10 |
|  |  | ‘Stressed’ | 12 |
|  |  | ‘A little anxious Concerned for patient’s well-being.’ | 15 |
|  |  | ‘Anxious and pressured to save the patients life but I try as much as possible to remain calm.’ | 16 |
|  |  | ‘It is an awful feeling because sometimes I tend to ask myself if I missed anything in my assessment of the patient.’ | 19 |
|  |  | ‘Concerned and increased adrenaline’ | 20 |
|  |  | ‘Worried for the patient…’ | 23 |
|  |  | Worried, exhilarated.’ | 24 |
|  |  | ‘Worried’ | 25 |
|  |  | ‘Concern for patient’ | 26 |
|  |  | ‘Obviously concerned for the patient, wanting to do the best fr then. Some anxiety depending on situation.’ | 27 |
|  |  | ‘Alert, concerned, sometimes on edge…’ | 30 |
|  |  | ‘My first focus is to try to work out what is going on but I am always concerned that I have missed something in my initial patient assessment at the start of the shift. I am anxious to provide the correct treatment in a timely fashion and am happy to start initiating treatment whilst waiting for medical review.’ | 33 |
|  |  | ‘Stressed, pressured’ | 34 |
|  | Stress aids cognition | ‘Adrenaline kicks in, I feel bad that I am often leaving my other patients to focus on this one’ | 1 |
|  |  | ‘At the time you are focused on doing the best for your patient but you do think if you’ve missed something that’s lead to this.’ | 5 |
|  |  | ‘Challenged but usually confident.’ | 8 |
|  |  | ‘I still remember now a patient who died when I was a student nurse and a young patient a few years ago. Sometimes the memories are hard, but it also tells me I still care and I believe pushes me to continue’ | 9 |
|  |  | ‘Worried for them. Enables me to do my utmost to ensure I provide a high quality of care in their best interest.’ | 11 |
|  |  | ‘It is an awful feeling because sometimes I tend to ask myself if I missed anything in my assessment of the patient…because I have that feeling at the back of my head therefore, I assess with the A-E assessment to prevent missing anything’ | 19 |
|  |  | ‘Under pressure, but I know what is required of me and I am able to stay calm…Yes, because I am able to carry out my assessment systematically and thoroughly.’ | 21 |
|  |  | ‘Focused, I become very direct and vocal in requesting support and delegating tasks to others.’ | 22 |
|  |  | ‘Maintaining a calm environment, easier to obtain factual assessment. When junior staff see this, helps them understand.’ | 26 |
|  |  | ‘Somewhat guilty , like I have missed something… I hope so, I hope it makes me more thorough’ | 29 |
|  |  | ‘concerned for patients welfare. Useful as I can put my knowledge and experience to good use…makes me more proactive, and confident.’ | 31 |
|  |  | ‘I stay calm and focus on patient’s needs and make sure nothing missed from the diagnosis and treatment…Panic kills both ways’ | 32 |
|  | Stress impedes clinical performance | ‘Not as confident. I personally would focus on the complaint at hand and based from trainings Ive gad they emphasize always an A—E Assessment but when in real scenario I just tend to dive straight on as to what the complaint is instead of assessing A-E’ | 6 |
|  |  | ‘Sometimes my thoughts race with the adrenaline and it might affect the systematic way of assessing.’ | 20 |
|  | Guilt of prioritising deteriorating patient | ‘Adrenaline kicks in, I feel bad that I am often leaving my other patients to focus on this one’ | 1 |
|  | Pragmatic prioritisation of deteriorating patient | ‘I feel bad that I am often leaving my other patients to focus on this one but I know that I am doing what is right and what is needed to give someone the best possible chance’ | 1 |
|  | Traumatising legacy | ‘I still remember now a patient who died when I was a student nurse and a young patient a few years ago. Sometimes the memories are hard, but it also tells me I still care and I believe pushes me to continue’ | 9 |
|  |  | ‘Of course, when you know that there is a probability of a patient dieing due to sudden detioration it will affect you emotionally and psychological but we are training to look calm, professional and coordinated at those moments.’ | 16 |
|  | Resignation to it being stressful | ‘No, I believe that it is a part of the profession that we have’ | 12 |
| 1. Nurses deliberately and empathetically tailor their actions | Deliberately tailoring scope of assessment | ‘I would probably focus on breathing at this stage as it where my concerns are.’ | 5 |
|  |  | ‘Mainly focuses directly an what is the current complaint.’ | 6 |
|  |  | ‘It would change from patient to patient’ | 12 |
|  |  | ‘Look at the overal picture of the patient’ | 19 |
|  |  | ‘Perform an assessment the type of which depends on how the patient is presenting I.e a-e, drabc, ecg etc.’ | 30 |
|  |  | ‘…spending a bit more time on any specific area that is a cause of concern (eg if SOB or low O2 SaO2, I would auscultate the chest to check for evidence of pulmonary oedema]…’ | 33 |
|  | Nurses as engaged and active actors | ‘…suggest and implement treatment…Use clinical judgement to escalate observation frequency…’’ | 1 |
|  |  | ‘…and assess for reversible causes…’ | 2 |
|  |  | ‘I’m also very passionate about providing safe and effective patient care…’ | 2 |
|  |  | ‘…put extra PVC in case for GTN infusion…’ | 6 |
|  |  | ‘There is 2 ways within my role 1. Within the heart attack service/cardiac arrest referral service I provide telephone support when requested or required for patients who have deteriorated on route and mobilize an appropriate response for when the patient arrives. For example, ambulance crew may call to advise a patients BP has dropped on route. If advice is required by the ambulance crew, this will be given, alongside assurance we are still ready to receive the patient to avoid the patient being diverted. 2. Face to face with patients on my ward, if I am concerned they have deteriorated, I assess what, where and how they are showing evidence of deterioration (physiological findings), also talk to the patient, to establish if they feel different, more unwell/better, but also use gut instinct. If something doesn't feel right with the patient, often the gut instinct tells your that. If I am concerned I will escalate primarily to the Cardiology SpR and the nurses involved in the patients care. Depending on what has changed I will also inform the Consultant Cardiologist if indicated/available. I work closely with Critical care outreach. If the change indicates or if immediate help is needed a medical emergency call will be made… When considering this question, I thought about how I teach other nurses to do patient assessment… I feel nurses are now being taught to look at numbers and warning scores and the use of gut instinct and simply looking a the patient has been lost. I see this when I am teaching and when conducting interviews… I feel I provide very good support and can give balanced constructive feedback… I still remember now a patient who died when I was a student nurse and a young patient a few years ago…Recently 3 staff approached me about a patient with sepsis who despite asking the SpR was not commenced on inotrope support and had no invasive monitoring for 24 hours. I listened to their concerns, completed an incident report and then completed a initial review of the patients care. I have submitted this for review with a Consultant Cardiologist as I agreed that there was a delay starting treatment…I am trying to influence how other staff assess their patients…For example, ambulance crew may call to advise a patients BP has dropped on route. If advice is required by the ambulance crew, this will be given, alongside assurance we are still ready to receive the patient to avoid the patient being diverted… Manage issues as you encounter them. If hypoxic, start oxygen.’ | 9 |
|  |  | ‘Yes, as being a nurse my responsibilty holds a impotant in my patient health… When my patient is deteriorating, i would like to help them better and try every possible reversible cause to improve my patient health.’ | 13 |
|  |  | Sometimes it is inevitable but for as long as I have done my best and necessary thing to save the patient i will feel fulfilled for doing everything | 14 |
|  |  | ‘I will start treatments such as IV fluids, IV analgesia , as prescribed and call the the doctor on duty.’ | 16 |
|  |  | ‘Depending on the result of my assessment, if I am able to intervene within my competency then I will. If beyond my own competency or needing further escalation, I will escalate to the SpR or consultant on call.’ | 17 |
|  |  | ‘When a patient deteriorates, I perform my assessment ensuring that I correct issues that could be reversed at that moment.’ | 19 |
|  |  | ‘Depending on clinical scenario- oxygen/ NIV/ I can perform ABG (extended role), patient positioning, IV access/ bloods. Cardiac monitoring/ ECG. Ensure patient is in a monitored bed space so patient is visible.’ | 27 |
|  |  | ‘If it is not my patient, I would review previous blood results and check on recent investigations / procedures to assess for complications and also review the drug chart to review prescriptions and any recent medications administered … Request ABG and CXR for patients with respiratory issues… I am anxious to provide the correct treatment in a timely fashion and am happy to start initiating treatment whilst waiting for medical review… I feel more comfortable in managing the situation if I have a very good second nurse working with me as I don't have to check up on everything or supervise everything myself… I do feel a responsibility to teach junior nurses in real time but usually have to do a debrief afterwards because it may not always be possible.’ | 33 |
|  | Unafraid of acknowledging limitations | ‘I’m also not afraid to ask questions, to understand rationale for decisions, to aid learning from experiences.’ | 2 |
|  |  | ‘…I am not afraid to ask for help when I need it.’ | 1 |
|  |  | ‘Limited clinical skills & knowledge.’ | 3 |
|  |  | ‘Undertake interventions to help improve patients condition within scope of practice’ | 5 |
|  |  | ‘Always wary of assuming I 'know it all' but not sure what I would add at this time’ | 8 |
|  |  | ‘I generally do feel very confident, but also know my limits and I know I have lost some of my skills since moving into a mixed clinical/managerial role. I know when to ask for help and who to ask and importantly ensuring the support comes.’ | 9 |
|  |  | ‘Not very much, as I am newly qualified and I always work with othe healthcare staff to get a wide range of opinions on my patients, as this will help me in making decisions.’ | 16 |
|  |  | ‘Depending on the result of my assessment, if I am able to intervene within my competency then I will. If beyond my own competency or needing further escalation, I will escalate to the SpR or consultant on call.’ | 17 |
|  |  | ‘A-E assessment and treat any immediate red flag as long as within my scope of practice.’ | 34 |
|  | Empathetic communication | ‘Tell the patient my concerns…’ | 5 |
|  |  | ‘Ensure patient informed and included, in this case language may be a barrier and access to interpretation may be beneficial’ | 8 |
|  |  | ‘…also talk to the patient, to establish if they feel different, more unwell/better…’ | 9 |
|  |  | ‘…communicate with patient…’ | 11 |
|  |  | ‘I think I complete the assessment all the time with patient's consent no matter how I feel, so I can offer an accurate description/diagnosis of what is happening. By doing this, I help in ensuring that the patients are presented with the most accurate information to help guide them come with an informed decision regarding their healthcare decisions.’ | 17 |
|  |  | ‘’ Talk to the patient, ask them how they are feeling, and reassure them’ | 29 |
| 1. Temporal dimensions of nurses’ assessments | Relevance of physiological trends/baseline | ‘Look back at normal trends…’ | 1 |
|  |  | ‘I also compare their baseline and their current state’ | 16 |
|  |  | ‘check baseline egg and compare to one taken at time of assessment. Track changes over time.’ | 31 |
|  |  | ‘…Track changes over time’ | 31 |
|  | Anticipating possible outcomes | Patients with STEMI especially anterior (LV) involvement, are at risk of pulmonary oedema, | 2 |
|  |  | ‘…in order for the patient to be treated in a timely and safe manner, improving outcomes.’ | 2 |
|  |  | ‘Start a check list for undergoing an angio.’ | 5 |
|  |  | ‘…you get to know the signs they exhibit that they are deteriorating and you can foresee the task and interventions that will be needed.’ | 6 |
|  |  | ‘Consider potential causes/ treatments and if able prepare next courses of action to prevent delays.’ | 27 |
|  | Patient history shapes assessment | ‘Obtain an ABG to assess oxygenation, to determine scale 1 or scale 2, given patients smoking history.’ | 2 |
|  |  | ‘Look at medical history’ | 15 |
|  |  | ‘The patient may be in the early stages of detiorition due to the fast breathing which may suggest that the body is compensating for a problem which may be due to his medical history and also there might be other underlying problems as patient is a smoker and has high cholesterol levels.’ | 16 |
|  |  | ‘…take a history then examine.’ | 30 |
|  | Urgency of situation | ‘…as soon as my patient shows signs of deterioration…’ | 2 |
|  |  | ‘…early Eacalation…’ | 3 |
|  |  | ‘Review by the doctor for urgent Angiogram’ | 7 |
|  |  | ‘Aware of risk that I delay escalation whilst completing my assessment.’ | 8 |
|  |  | ‘Recently 3 staff approached me about a patient with sepsis who despite asking the SpR was not commenced on inotrope support and had no invasive monitoring for 24 hours. I listened to their concerns, completed an incident report and then completed a initial review of the patients care. I have submitted this for review with a Consultant Cardiologist as I agreed that there was a delay starting treatment.’ | 9 |
|  |  | ‘Confident enough to identify and do escalation in a swift and timely manner.’ | 12 |
|  |  | ‘I will escalate to the medical team as soon as possible…To escalate in timely manner will reduce thw emergency situation’ | 13 |
|  |  | ‘Proper escalation and early detection is very essential’ | 14 |
|  |  | ‘I feel sorry for what they are going through, but I ensure that their needs are met by our timely response to their conditions.’ | 17 |
|  |  | ‘However, I ensure that my assessment does not delay getting help for the patient from the medical team and other teams such as CCOT.’ | 19 |
|  |  | ‘I feel like I need to be proactive and get this sorted asap.’ | 28 |
|  |  | ‘if they deteriorate suddenly and unexpectedly Medical Alert call for the urgent assessment.’ | 32 |
|  |  | ‘I am anxious to provide the correct treatment in a timely fashion and am happy to start initiating treatment whilst waiting for medical review.’ | 33 |
|  |  | ‘Refer immediately to doctors as well as CCOT’ | 34 |
|  | Repeat assessment | ‘Repeat gas/obs and reassess patient.’ | 3 |
|  |  | ‘I am therefore always undertaking quick A-Es certainly when first meeting patients.’ | 5 |
|  |  | ‘Refer immediately to doctors as well as CCOT. Re-assess’ | 34 |
| 1. What makes nurses’ confident in the scope of their assessments. | Experience cultivates confidence | ‘This is probably due becoming a more confident practitioner as I’ve progressed and gained more experience through my career.’ | 2 |
|  |  | ‘I just started working in the Coronary Care Unit, so I am still working on my confidence’ | 7 |
|  |  | Very confident, nursing for 38 years with over 10 years of CCU. Also GIC and ALS Instructor. Previously an ALERT Instructor. 28 Years as a nurse in the Royal Naval includes Battlefield Advanced Trauma Life Support Training and practical experience in the Battlespace including working as a lone medic. | 8 |
|  |  | ‘I am very efficient at interpreting clinical findings, including ECG's and have a solid knowledge of blood gas interpretation. I think this all comes from 28 years of experience in cardiac nursing.’ | 9 |
|  |  | ‘Quite confident . Experience as a senior nurse. ALS trained . ECG measurement and interpretation trained.’ | 11 |
|  |  | ‘As being working in CCU and HAS nurse has helped me to assess deteriorating patients.’ | 13 |
|  |  | Pretty confident as I’ve worked in CCU for many years | 15 |
|  |  | ‘I am quite confident after having been in Cardiology/CCU/Cathlab for almost a decade.’ | 17 |
|  |  | ‘In the beginning was getting anxious to see if any patients deteriorates , gradually with experience made me more confidence and keen to learn.’ | 18 |
|  |  | ‘I feel 100% confident assessing deteriorating patients due to experience and the availability of additional support from nursing colleagues and medical team.’ | 20 |
|  |  | ‘I feel confident having worked in ICU’ | 21 |
|  |  | ‘Confident, as over 30 years in cardiology.’ | 26 |
|  |  | ‘Confident due to experience’ | 27 |
|  |  | ‘Fairly confident due to experience working in CCU where patients have the tendency to deteriorate often’ | 29 |
|  |  | ‘Pretty confident, I've been doing it for a long time in high pressure environments where patients are often acutely unwell…’ | 30 |
|  |  | ‘Experience of working both in CCU, on cardiology/general wards, pre-hospitably and in ICU has given me a breadth of skills and experience of assessing and treating deteriorating patients.’ | 31 |
|  |  | ‘Reasonably confident with 22 years nursing experience.’ | 32 |
|  |  | ‘I feel confident in accessing deteriorating patients as I have over 20 years experience in CCU nursing and have worked with, and learned from, many nursing and medical experts in the speciality over the years. I have also undertaken university modules in history taking and physical examination of the adult which included mentorship and assessment by a consultant cardiologist. I have worked as a chest pain nurse specialist, a role that required working with a large degree of autonomy. I also enjoy a good relationship with the SpRs and consultants, based on mutual professional respect, and know that they will review a patient rapidly if I am concerned… To a certain degree. I feel the scope of my patient assessments is driven by experience, learning from previous omissions / errors and a desire to do the best for my patients. It makes going home easier’ | 33 |
|  |  | ‘Fairly confident. From experience in CCU.’ | 34 |
|  | Education legitimises practise | ‘A B C D E approach as taught in my ALS course.’ | 1 |
|  | Education legitimises confidence | ‘Very confident, nursing for 38 years with over 10 years of CCU. Also GIC and ALS Instructor. Previously an ALERT Instructor. 28 Years as a nurse in the Royal Naval includes Battlefield Advanced Trauma Life Support Training and practical experience in the Battlespace including working as a lone medic.’ | 8 |
|  |  | ‘Quite confident . Experience as a senior nurse. ALS trained . ECG measurement and interpretation trained.’ | 11 |
|  |  | ‘I have also completed a degree in cardiology and finished advanced assessment modules both in full physical assessment and focused cardio/respiratory assessment. All of which are helpful in my confidence level.’ | 17 |
|  |  | ‘Confident. ALS provider and ACP with appropriate skill set.’ | 24 |
|  |  | ‘Confident due to…ALS provider.’ | 27 |
|  |  | ‘Pretty confident, I've been doing it for a long time in high pressure environments where patients are often acutely unwell and have advanced training to support me.’ | 30 |
|  |  | ‘I have completed numerous extra curricular training opportunities to also further enhance these experiences and improve m y confidence.’ | 30 |
|  |  | ‘I have also undertaken university modules in history taking and physical examination of the adult which included mentorship and assessment by a consultant cardiologist.’ | 31 |
| 1. What nurses believe would improve the scope of their assessments. | Education to improve practise | ’Simulation in house’ | 1 |
|  |  | ‘More training - Chest Auscultation, ALS, Bipap.’ | 3 |
|  |  | ‘I do feel I perhaps could learn some more advanced skills to undertake better assessments…. I need more skills and regular sessions on abgs, ecmo.’ | 5 |
|  |  | ‘Further training on management of patients in Coronary Care Unit’ | 7 |
|  |  | ‘If others haven't done them- I will suggest formal training in physical assessment. In my unit, all of our nurses go through a development process, helping them gain experience and familiarity in cardiac conditions, exams, and testing using a guided development book encouraging them to take ownership of their development with assistance of the program and their designated mentors. We also fund them to complete various university modules in ECG, arrhythmia, ACS and HF. Some of them have competed a full degree.’ | 17 |
|  |  | ‘…education’ | 21 |
|  |  | ‘Everyone should attend courses on recognising deteriorating patients.’ | 22 |
|  |  | ‘Ongoing training on life support.’ | 23 |
|  |  | ‘…education, further simulation training..’ | 22 |
|  |  | ‘nurse educators going through specific common scenarios in the wet lab situation to run through how to assess, treat and manage these patients.’ | 31 |
|  |  | ‘Continuous knowledge/skills update’ | 34 |
|  | Infrastructure to improve practise | ‘Staffing level’ | 4 |
|  |  | ‘Having paper charts was much more effective for me as a quick visual guide. Having the NEWS 2 parameters printed out as a visual guide.‘ | 5 |
|  |  | ‘Blood gas on ward’ | 10 |
|  |  | ‘To have more time. Made possible with better staffing.’ | 15 |
|  |  | ‘Better staffing.’ | 20 |
|  |  | ‘Safer staffing ratios.’ | 27 |
|  | Experience to improve practice | ‘Time in ICU…’ | 21 |
|  |  | ‘Being back at a tertiary centre - get rusty at a DGH.’ | 24 |
|  |  | ‘Auscultation skills are easily lost if not practised regularly and are beyond the scope of practice of most of the CCU nursing team.’ | 33 |
|  | Mistakes as a learning opportunity | ‘To a point, in terms of learning from your mistakes’ | 5 |
|  |  | Recently 3 staff approached me about a patient with sepsis who despite asking the SpR was not commenced on inotrope support and had no invasive monitoring for 24 hours. I listened to their concerns, completed an incident report and then completed a initial review of the patients care. I have submitted this for review with a Consultant Cardiologist as I agreed that there was a delay starting treatment.’ | 9 |
|  |  | ‘To a certain degree. I feel the scope of my patient assessments is driven by experience, learning from previous omissions / errors and a desire to do the best for my patients. It makes going home easier.’ | 33 |
|  | Caring for deteriorating patients is a learning opportunity | ‘Yes. The more I get to look after unwell patients, the more it is an opportunity to learn. It’s about having the right mindset.’ | 6 |
|  | Nurses communicating up the hierarchy | ‘…I find the challenge getting certain people from ccot to take referrals seriously.’ | 5 |
|  |  | ‘Just the confidence of escalating to the medical team.’ | 6 |
| 1. Use of NEWS | Use of NEWS described | ‘…1hour or 15 minutes observation depending on patient news score’ | 4 |
|  |  | ‘Perform observations and frequency on NEWS’ | 6 |
|  |  | ‘…refer in accordance with NEWS / local guidelines…’ | 8 |
|  |  | ‘Observation every 4-6 hourly unless patients condition changes.’ | 14 |
|  |  | ‘As per NEWS which is 3’ | 34 |
|  |  | ‘Obtain an ABG to assess oxygenation, to determine scale 1 or scale 2, given patients smoking history.’ | 2 |
|  | Use of NEWS discussed | ‘I believe further ABG interpretation training should be given, as there is a big misconception about oxygenation especially around COPD patients and automatically defaulting to Scale 2 without evidence and justification, meaning we’re seeing hypoxic patients, that are missing initiation on CPAP.’ | 2 |
|  |  | ‘Assess physiological observations but also look beyond the observations numbers. Skin colour, skin temperature. Is it normal or are they cold and clammy… I feel nurses are now being taught to look at numbers and warning scores and the use of gut instinct and simply looking a the patient has been lost. I see this when I am teaching and when conducting interviews.’ | 9 |
|  |  | ‘Better training for junior staff in how to recognise a deteriorating patient (without nursing by numbers, ie using SEND or NEWS which are wholly unsuitable for patients in a coronary care unit environment’ | 33 |
